# Metabolomic and proteomic investigations of impacts of titanium dioxide nanoparticles on *Escherichia coli*

**DOI:** 10.1371/journal.pone.0178437

**Published:** 2017-06-01

**Authors:** Mariane Planchon, Thibaut Léger, Olivier Spalla, Gaspard Huber, Roselyne Ferrari

**Affiliations:** 1 NIMBE, CEA, CNRS, Université Paris-Saclay, CEA Saclay 91191 Gif-sur-Yvette, France; 2 Université Paris Diderot, Sorbonne Paris Cité, IPGP, UMR 7154, Paris Cedex 13 France; 3 iCEINT, International Consortium for the Environmental Implications of Nanotechnology; 4 Mass Spectrometry Laboratory, Institut Jacques Monod, UMR 7592, Univ Paris Diderot, CNRS, Sorbonne Paris Cité, Paris, France; 5 Université Paris Diderot, Sorbonne Paris Cité, LIED, UMR 8236, Paris, France; VIT University, INDIA

## Abstract

In a previous study, it was demonstrated that the toxic impact of titanium dioxide nanoparticles on *Escherichia coli* starts at 10 ppm and is closely related to the presence of little aggregates. It was also assumed that only a part of the bacterial population is able to adapt to this stress and attempts to survive. Proteomic analyses, supported by results from metabolomics, reveal that exposure of *E*. *coli* to nano-TiO_2_ induces two main effects on bacterial metabolism: firstly, the up-regulation of proteins and the increase of metabolites related to energy and growth metabolism; secondly, the down-regulation of other proteins resulting in an increase of metabolites, particularly amino acids. Some proteins, e.g. chaperonin 1 or isocitrate dehydrogenase, and some metabolites, e.g. phenylalanine or valine, might be used as biomarkers of nanoparticles stress. Astonishingly, the ATP content gradually rises in relation with the nano-TiO_2_ concentration in the medium, indicating a dramatic release of ATP by the damaged cells. These apparently contradictory results accredit the thesis of a heterogeneity of the bacterial population. This heterogeneity is also confirmed by SEM images which show that while some bacteria are fully covered by nano-TiO_2_, the major part of the bacterial population remains free from nanoparticles, resulting in a difference of proteome and metabolome. The use of combined–omics has allowed to better understand the heterogeneous bacterial response to nano-TiO_2_ stress due to heterogeneous contacts between the protagonists under environmental conditions.

## Introduction

Environmental pollution by manufactured nanoparticles is receiving more and more attention as the potential accumulation in soil and water compartments of nanoparticles could interact with organisms and affect human health problems [1]. Toxicological and eco-toxicological studies of nanomaterials [1–3], more particularly nano-TiO_2_, on several models such as plants [4], microorganisms [5–8] and animals [9–11], have already been performed. In a previous work [12], we reported weak bactericidal effects of nano-TiO_2_ towards *E*. *coli* in Seine river fresh water–a natural water that provides a better toxicity study medium than ultrapure water or cell culture broth. However, investigations of detailed mechanisms on (i) how nano-TiO_2_ produce their toxic effects in biological systems and on (ii) how organisms acclimatize themselves in response to stress from nanoparticles are still scarce. The metabolic responses of cells and underlying molecular mechanisms against various nano-TiO_2_ are not yet completely understood. The present study is intended to shed some light on these mechanisms, based on metabolomics and proteomics analyses.

The metabolome is defined as the quantitative collection of the low-molecular-weight molecules (metabolites) required for growth and proper functioning of a cell [13]. According to this definition, metabolomics is the characterization and quantification of the cellular metabolome. It provides a snapshot of the metabolic status of a cell in a particular physiological condition [14]. Because it is a non-selective method, able to detect and quantify simultaneously virtually any metabolite, and despite its low sensitivity, Nuclear Magnetic Resonance (NMR) spectroscopy is widely used in metabolomics [15,16], in particular when *E*. *coli* is concerned [17–19]. The proteome analysis brings an additional information as it enables measurement of whole-protein (enzyme) expression levels, facilitating the construction of metabolic pathways [20]. In particular, proteomic analysis highlights the effect of stress on metabolic pathways necessary to maintain the energy homeostasis within the cells. Under stress conditions, *E*. *coli* expresses metabolic pathways with relatively inexpensive proteome synthesis requirements, instead of using more efficient, but also more expensive anabolic pathways [21]. Proteomic analysis of heat shocked *E*. *coli* cultivated in bioreactors revealed the complexity of their metabolism [22]. Since years, this Gram-negative bacillus has become one of the most popular models used for studying roles of metal or environmental stress thanks to its rapid duplication time and response to toxics [23]. *E*. *coli*’s abundance in surface waters polluted by anthropic activities makes it a suitable candidate for environmental nanotoxicology [12]. Nano-TiO_2_ are produced at a large scale for applications [24] in paints [25], cosmetics and sunscreens [26], food additives [27] and photocatalytic water purification systems [28]. Without photoactivation, nano-TiO_2_ is considered chemically inert but it has been reported to exhibit a strong photocatalytic and antibacterial activity under UV_A_ irradiation *via* the production of reactive oxygen species (ROS) [29,30]. Some studies, including our previous results [12], reported a moderate toxicity of nano-TiO_2_ towards bacteria even in the absence of UV illumination [31,32]. It was observed that the addition of NaCl (50, 200 mg L^-1^) reduced the toxicity of nano-TiO_2_ towards *E*. *coli* [33]. However, as far as we know, the effects of nano-TiO_2_ on the metabolome or the proteome expression of *E*. *coli* had never been reported yet.

In the present study, analyses of both proteome and intracellular water-soluble metabolome of *E*. *coli* strain MG1655 when exposed to effective dose of various nano-TiO_2_ under ultra-violet irradiation or under normal light are carried out. Up- and down-regulation of differentially expressed proteins and metabolites have been investigated. Mechanisms of cellular damage or adaptation against different kinds of nano-TiO_2_ are finally proposed.

## Materials and methods

### Preparation of the nanoparticles and description of the Seine river natural medium

Four types of nano-TiO_2_ were used. Three of them (called R for rutile, M for mixed and A for anatase) were synthesized following classical recipes [34] while the fourth nanoparticle, P25, was obtained from Degussa (Evonik, Germany). For each powder, aliquots (100 mg) were ultrasonicated (Sonicator Sonics & Materials, VCX 500-220V, 500 W, 20 kHz, 13 mm disruptor horn, Connecticut) for 10 min at 20 W. This operation produced suspensions containing 5000 ppm in mass fraction. Extemporaneously before being in contact with the bacterial cells, an aliquot of this freshly prepared high concentrated stock suspension of nano-TiO_2_ was dispersed by ultrasonication and then diluted at the desired concentration in the Seine river fresh water. The use of ultrasounds allowed breaking micrometric aggregates, so the mean sizes of nano-TiO_2_ aggregates in aqueous suspension was below 1 μm. The characterization of the four nano-TiO_2_ was carried out and is summarized in S1 Table in supplemental material. The hydrodynamic size of nano-TiO_2_ aggregates in the Seine river water was determined with Dynamic Light Scattering (Malvern nanosizer) which allowed size determination below 1 μm. Cryo-TEM (cryo-Transmission Electron Microscopy) was performed on the different samples in order to examine the aggregate sizes. This technique was used to avoid the classical TEM drying artifacts. Nano-TiO_2_’s electrophoretic mobilities were measured after dissolution in Seine river fresh water. Zeta potential values were calculated as described in Planchon et al. [12].

The Seine river samples were freshly collected from the Seine River near Paris Diderot University at the Quai Saint Bernard. The physicochemical parameters of the water were measured immediately after the collection before the water was filtered on 0.22 μm pore size membranes (cellulose acetate, Millipore) to remove all the indigenous microorganisms larger than 0.22 μm. The filtered water was stored at 4°C for experiments and constitute our reference interaction medium. The content of dissolved Ti (IV) was also analyzed by Inductively Coupled Plasma Atomic Emission Spectroscopy and found to be below 1 nM.

### Bacterial strains and growth conditions

Although indigenous *E*. *coli* is already present in Seine River water in a significant concentration (2000 cells/100 mL measured with MUG microplates from Bio-Rad France), we choose to use *E*. *coli* wild type strain MG1655 which corresponds to the wild type (F^-^λ^-^) frequently used as a reference in genome sequencing. Our model strain was supplied by Dr. Dukan (UMR 9043, France). Before the exposition to the nano-TiO_2_, MG1655 was grown overnight in Luria Bertani medium at 37°C aerobically under vigorous shaking (122 rpm). The overnight culture was then diluted to an absorbance at 600 nm (OD_600_) of 0.1 ± 0.02 in 50 ml fresh medium and cultivated in the same conditions to reach exponential phase (OD_600_ = 0.325 ± 0.02). Cells were harvested by centrifugation at 4500 rpm for 10 min (Eppendorf 5810 R), washed twice with Seine River fresh water and suspended in the same medium at a concentration of 1.5 10^7^ cells/ml (OD_600_ = 2.5).

Depending on the method analysis, aliquots of this culture were submitted to the four nano-TiO_2_ stock suspensions to obtain exposure concentrations of massic fraction of 10 ppm, i.e. the lowest limit of toxicity as determined by Planchon *et al*. [12], and 100 ppm, far above this limit. The contact was performed with continuous orbital shaking (122 rpm) to avoid the precipitation of the nano-TiO_2_ at 37°C under room light (Neon 4000K white neutral, average power in UV_A_ of 1.5 10^−3^ mW/cm^2^) or under UV_A_ irradiation (VL-6LC, 365 nm, 80 mW/cm^2^) for 3 hours. It was previously reported that the survival fraction of bacteria does not significantly differ under dark or room light conditions [12]. The chosen exposure time corresponds to the time of a stable suspension of the nano-TiO_2_ in Seine river water medium and to the survival time of bacteria. After 4 hours, virtually all bacteria were dead [12] due to the very low nutriments contents of the medium, which allows only a short survival of bacterial cells.

### Extraction of proteins and metabolites

After cell contact with 0, 10 or 100 ppm nano-TiO_2_ under room light, the extraction of proteins was performed by total lysis of bacterial cells. To do so, bacterial suspension treated with nano-TiO_2_ were harvested by centrifugation at 4°C, 9600 rpm for 5 min. Pellets were then suspended in sterile ultrapure water with 1% SDS (Sodium Dodecyl Sulfate). Noteworthy point, the SDS extraction of total proteins for proteomic analysis was recognized to be very efficient [35]. After 5 min at 100°C, suspensions were harvested again by centrifugation (10 min, 14 000 rpm) at room temperature and supernatants containing released proteins during cell lysis were collected and kept at -20°C until Inductively Coupled Plasma Mass Spectrometry analysis.

The metabolite extraction was performed after cell contact with 0, 10 or 100 ppm nano-TiO_2_ under room light or UV_A_ irradiation (365 ± 25 nm, with a surface power density of 30 mW/cm^2^ or 80 mW/cm^2^). Bacterial suspension treated with nano-TiO_2_ were harvested by centrifugation (5 min, 6000 rpm, 4°C). Pellets were then suspended in perchloric acid and boiled to trigger cell lysis and metabolite release. This extraction was optimized by testing different durations of ebullition and different concentrations of acid. Suspensions were then harvested by centrifugation (10 min, 10 000 rpm) and the supernatant that contains the water soluble metabolites were collected and kept at -20°C until ^1^H NMR analysis. Each experiment was triplicated.

### NMR acquisition and metabolomics data analysis

A 720 μl aliquot of sample was loaded into an NMR tube (HL5-7, CortecNet) for analysis and 80 μL of deuterated water were added for signal locking. ^1^H NMR spectra were obtained on a DRX600 Bruker Avance II spectrometer operating at 599.91 MHz equipped with a 5-mm inverse detection cryogenic probe. For all samples, ^1^H NMR spectra were acquired at 20°C using water suppression by excitation sculpting with pulsed field gradients [36]. 256 transients were collected into 32768 data points for each spectrum with a spectral width of 16 ppm, a recycle delay of 1.5 s and an acquisition time of 1.7 s. A π/2-shifted sine-squared function was applied to all free induction decays prior to Fourier transformation giving 32768 data points in the transformed spectra. Spectra were individually phased and then base-line corrected by a constant.

For data analysis, 136 and 180 areas were defined for series of experiments aiming at describing the influence of (i) nanoparticle type and concentration and (ii) nanoparticle type and ultra-violet irradiation intensity, respectively. An example of region definition is shown in S1 Fig. While defining these areas, a slight chemical shift variations mainly originating from slight pH variation was taken into account. Therefore, areas are defined in order to contain signals that seem to be assigned to the same metabolites or classes of metabolites. Also multiplets and signals whose individual integration is not easy are clustered in the same areas. The region of the residual proton signal of deuterated water was discarded. Spectra were exported to Excel software prior to an integration step of each area that consists in subtracting from the sum of individual intensities of points belonging to a given area, the mean of the intensity of the 2 points bordering the area and their 4 closest points, multiplied by the width of the area (S1 Fig). This procedure helps at removing components coming from large signals when sharp areas are defined. Taking the average of three intensities instead of one on the right and on the left of each area as references decreases experimental errors. The spectra were consecutively normalized to the total sum of the spectral integrals to compensate for sample concentration differences. A Pareto scaling was chosen as it was shown to be more appropriate for analytical spectroscopic data [37]. A Principal Component Analysis (PCA) was performed using MS-DIAL [38].

### Proteomic analysis by mass spectrometry

Samples were digested overnight at 37°C by sequencing grade trypsin (12.5 μg/ml, Promega Madison, Wi, USA) in 20 μl of NH_4_HCO_3_ (25 mM) and 10% acetonitrile. Digests were analyzed by a LTQ Velos Orbitrap (Thermo Fisher Scientific, San Jose, Ca, USA) coupled to a nano-LC RSLC system (Thermo Fisher Scientific, San Jose, Ca, USA). Chromatographic separation of peptides was performed with the following parameters: column Acclaim Pepmap RSLC C18 (25 cm, 75 μm i.d., 100 A), 300 μl/min flow, gradient rising from 98% solvent A (water, 0.1% formic acid) to 35% B (acetonitrile, 0.1% formic acid) in 100 min, then to 80% B in 4 min and finally to 2% B in 2 min. Peptides were analyzed in the orbitrap in full ion scan mode at a resolution of 30 000 and a mass range of 400–1800 m/z. Fragments were obtained with a collision-induced dissociation (CID) activation with a collisional energy of 40%, an activation Q of 0.250 for 10 ms, and analyzed in the LTQ. MS/MS data were acquired in a data dependent mode in which the 20 most intense precursor ions were isolated, with a dynamic exclusion of 20 seconds and an exclusion mass width of 10 ppm. Data were processed with Proteome Discoverer 1.3 software (Thermo Fisher scientific, San Jose, CA) coupled to an in house Mascot search server (Matrix Science, Boston, MA; version 2.3.02). The mass tolerance of fragment ions was set to 10 ppm for precursor ions and 0.6 Da for fragments. The following variable modifications (2 maximum per peptide) were allowed: oxidation (M), phosphorylation (STY). The maximum number of missed cleavages was limited to 2 for trypsin digestion. MS-MS data were searched against NCBI nr databases with the *E*. *coli* taxonomy. A reversed database approach was used for the False Discovery Rate estimation (FDR). A threshold of 1% was chosen for this rate. The scores obtained by this procedure were used in the following analysis.

As both the difference and the ratio between scores are valuable parameters to discriminate the samples, a unique parameter has been calculated for each protein in each sample. For each protein, nanoparticle type and concentration, the difference and the ratio between scores in the presence and the absence of the nanoparticles has been calculated, as soon as these scores has been obtained from the mass spectrometry and FDR analysis. In order to give the same attention to difference and ratio values, these values were weighted by the reverse of their standard deviation over the values obtained for all nano-TiO_2_ types and concentrations. The sum of the weighted values was used as a unique averaged integrated score describing the evolution of concentration of proteins in arbitrary units and in a qualitative way.

### Adenosine-5'-triphosphate (ATP) assay in the medium

Exponentially growing *E*. *coli* cells were resuspended in Seine river fresh water and incubated at 37°C in a shaking bath (122 rpm) for 3 h with 0, 50 or 100 ppm of P25 nano-TiO_2_ under room light. ATP release in the samples was determined by a luciferase-luciferin enzymatic assay kit BacTiter-Glo^TM^ from Promega in a microplate reader (Infinite 2000, Tecan). All experiments were done in triplicates in 96 well-microplates for chemiluminescence (Eppendorf). The BacTiter-Glo^TM^ reagent is directly added to bacterial cells in medium and triggers cell lysis and ATP recovery at the same time. The luminescence can be measured without washing cells or removing the medium. A series of blank as well as control are realized on Seine river fresh water and bacterial cells without nano-TiO_2_, respectively. Determination of ATP concentration in the medium was performed using a standard curve established on serial dilutions of ATP as recommended by Promega.

## Results

### Reproducibility and optimization of metabolite extraction protocol

The reproducibility was assessed by duplicating the sample preparation from the perchloric acid extraction protocol, also by triplicating spectra for most experiments including the analysis of the four types and the two concentrations of nanoparticles under different conditions of illumination. NMR spectra recorded for sample obtained in the same conditions were much more similar than spectra obtained for samples in different conditions.

**Fig 1:**
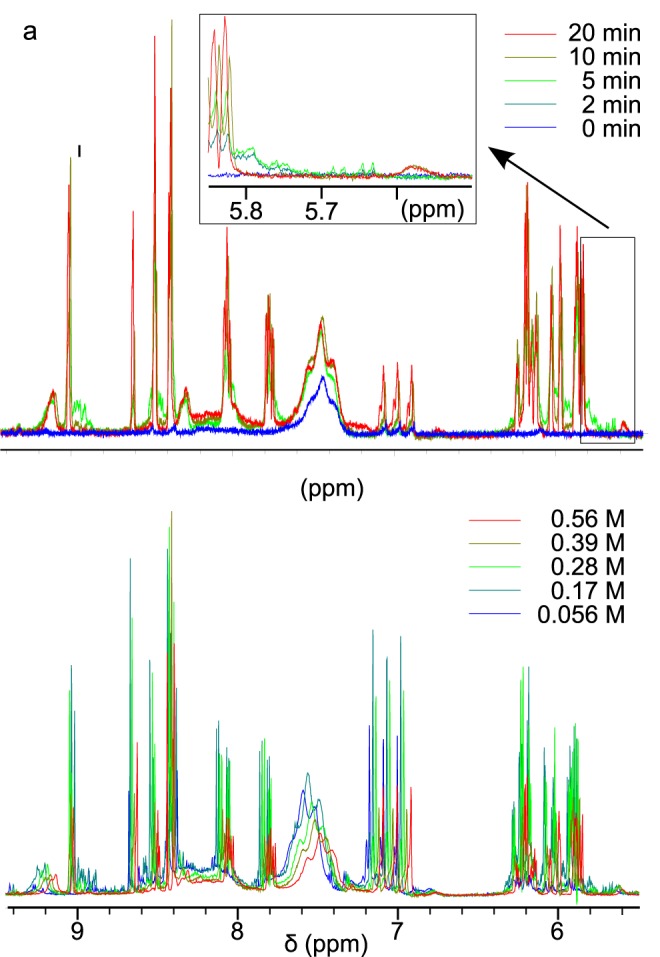
Comparison of the downfield part of NMR ^1^H spectra of *E*. *coli* metabolites (upfield part in supplemental material), after 3 h incubation in Seine river water under room light, with different extraction protocols. (a) influence of boiling time for cell lysis: no ebullition (deep blue) and 2 to 20 min ebullition (cyan to red) in a 0.56 M perchloric acid solution; (b) influence of perchloric acid concentration: 0.056 to 0.56 M (blue to red), with a boiling time of 10 min. The arrow points out areas where decreasing intensity at long boiling time is an indicator of the degradation of some metabolites.

The metabolite extraction protocol was optimized by varying the boiling time and the perchloric acid concentration (Fig 1, S2 and S3 Figs). We noticed that most NMR signals increased with boiling time, while a few decreased, such as in the 5.75–5.80 ppm region, which was probably due to degradation. Ten minutes was the boiling time retained. The amount of metabolites extracted was globally improved by acidifying the solution with higher perchloric acid concentrations, but we noticed a phenomenon of degradation for some metabolites at high perchloric acid concentration, for example around 7.5 ppm. A perchloric acid concentration of 0.17 M was thus chosen as a compromise between acidic extraction and degradation.

### Metabolome analysis

**Fig 2:**
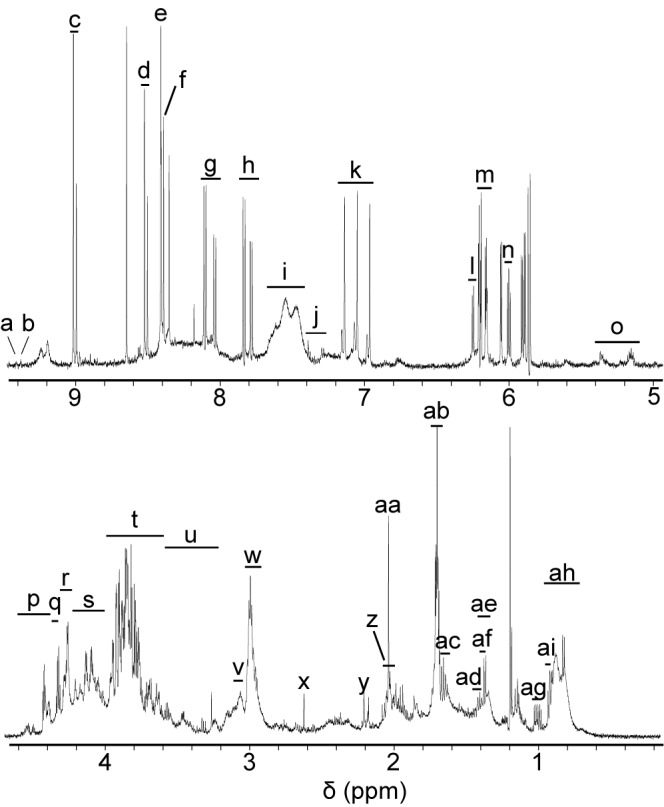
One dimensional 600 MHz ^1^H NMR of an extract of *E*. *coli* not stressed by nano-TiO_2_ and incubated under room light. Resonance assignments are given in S2 Table. Code number: a- NADP+; b- NAD+; c&n- 5'GMP; d- 5'-AXP; e- 5',3'-cAMP; f- 5',3'-cAMP; g- 5'-CMP or 5'-dCMP; h- 5'-UMP or UDP-glucose; i- NH3+ moieties, mainly putrescine, also cadaverine and ornithine; j- phenylalanine; k- ammonium; l- 5'-AXP & 5',3'-cAMP; m- 5'-CMP and 5'-dCMP; o&t- carbohydrates & amino acids; p- nucleoside derivatives, carbohydrates and amino acids; q- mainly 5'-GMP; r- mainly lactate, also nucleoside derivatives, carbohydrates and amino acids; s- nucleoside derivatives, carbohydrates and amino acids; u- carbohydrates; v- ornithine; w- main putrescine, minor cadaverine and ornithine; x- succinate; y- oxaloacetate; z- ornithine; aa- mainly acetate, also ornithine; ab- mainly putrescine, also ornithine; ac- arginine, lysine and cadaverine; ad&af- sharp signals: arginine, lysine and cadaverine; ae- broad signals: lactate; ag-valine; ah- broad signals: fatty acids; ai- leucine.

The typical one dimensional ^1^H NMR spectra of the *E*. *coli* extracts for the control case (no exposition to nano-TiO_2_) is shown in Fig 2. Those spectra comprise many hundreds of peaks. The resonances were tentatively assigned to specific metabolites according to TOCSY NMR spectroscopy and to the literature data available at pH 3 [17]. A total of 32 metabolites or families of metabolites were identified including amino acids (e.g. phenylalanine and valine), organic acids (e.g. acetate, lactate) and nucleotides (e.g. NAD, NADP) (Fig 2 and S2 Table). There are no dramatic spectral changes among the different exposition conditions and with the presence of nanoparticles, except peak intensity alterations, which reflect variations in metabolite concentrations. The variations of peak intensity were investigated by PCA.

**Fig 3:**
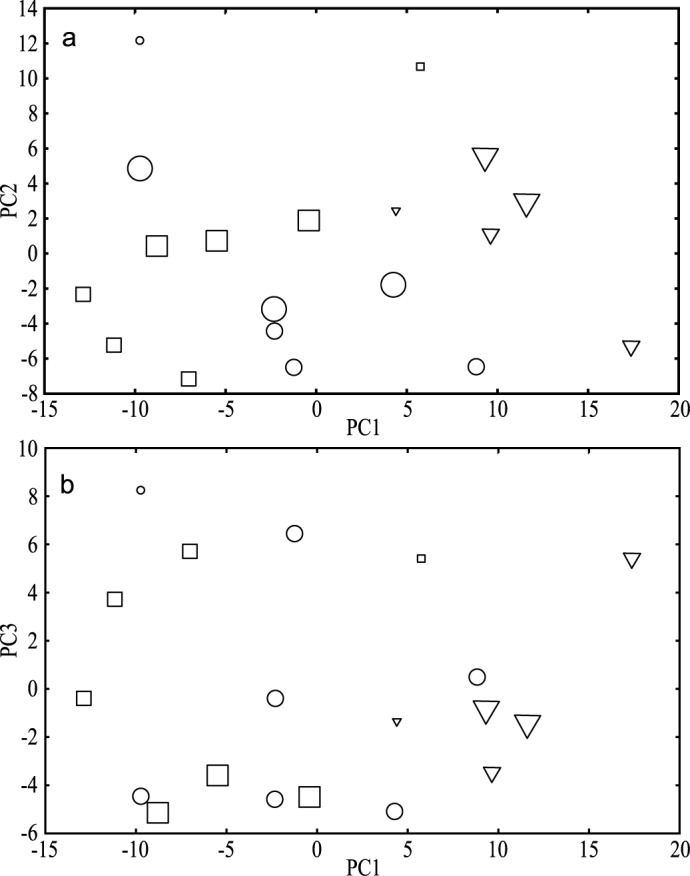
Score plots from the PCA of replicates of *E*. *coli* MG1655 nano-TiO_2_ stressed according to the type of nano-TiO_2_ and conditions of illumination with 100 ppm of nano-TiO_2_. R, P25 and control are coded in circles, squares and triangles, respectively. Room-light, 30 mW/cm^2^ and 80 mW/cm^2^ illumination are represented by small, average and large symbols, respectively. (a) Principal components 2 *vs*. 1. (b) Principal components 3 *vs*. 1.

Score plots from the PCA obtained by varying the type and the conditions of illumination are shown in Fig 3. Experiments with the same conditions are generally well clustered, with the exception of R4 illuminated at 80 mW/cm^2^, showing the reliability of the analysis. The three first principal components, PC1, PC2 and PC3, represent 41%, 16% and 11% of the dispersion, respectively. PC1 tends to discriminate control (strong positive values) *vs* nano-TiO_2_ treatment of the cells. PC2 and PC3 underscore the distinction between samples with or without illumination particularly when nanoparticles are present. This suggests that the influence of UV_A_ on metabolic profile of *E*. *coli* was amplified by the presence of nano-TiO_2_. One reason could be that the production of ROS is exacerbated by nano-TiO_2_ only under photocatalytic conditions, whereas bacterial metabolism is not strongly altered by UV_A_ alone or by nano-TiO_2_ alone [12].

**Fig 4:**
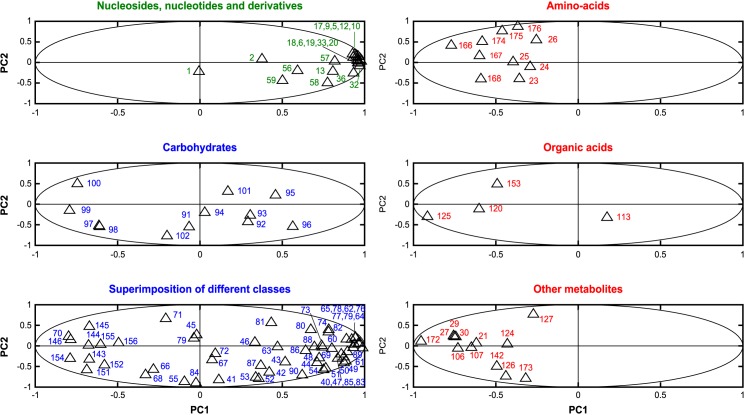
Loading plots from the PCA of replicates of *E*. *coli* nano-TiO_2_ stressed according to the type of nano-TiO_2_ and to the conditions of illumination with 100 ppm of nano-TiO_2_. Only assigned areas are shown (see S2 Table), and split on graphs for a better readability.

The loading plots from the PCA analysis are shown in Fig 4 which reports that some families of metabolites related to specific metabolism pathways are globally influenced by the presence of nano-TiO_2_. For example, the classes comprising nucleosides, nucleotides and analogues, identified on the basis of TOCSY correlations, see S2 Table, exhibit for almost all of them (except for NADP) a positive projection on PC1 (upper left panel of Fig 4). All the intense and sharp unassigned signals in the 9.3–7.7 and 6.3–5.8 ppm, where signals characteristic of nucleosides, nucleotides and analogs are observed, exhibited the same tendency. Signals of carbohydrates are dispersed left and right onto PC1 axis (middle left panel of Fig 4). Finally, signals of amino acids, of most of the organic acids and of other types of metabolites exhibit a negative projection on PC1 (right panels of Fig 4).

### Proteome analysis

**Fig 5:**
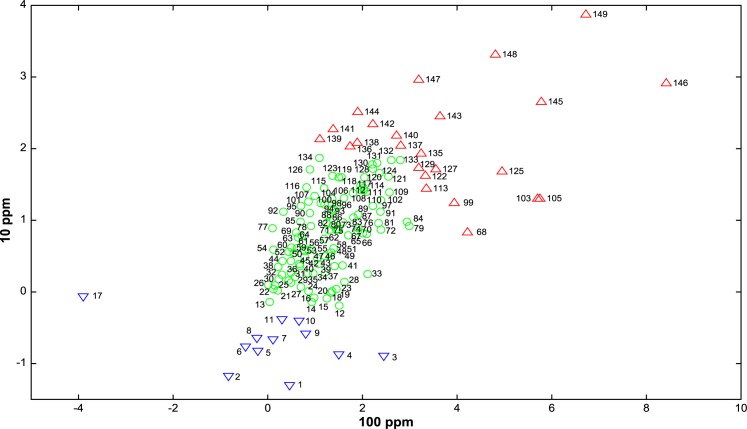
Averaged integrated score of proteins extracted from total lysis of *E*. *coli* culture in contact with 10 ppm nano-TiO_2_ as a function of the averaged integrated score when cells are in contact with 100 ppm nano-TiO_2_. The average figures are computed based on all four nano-TiO_2_ treatments. Proteins in red triangles pointing upwards are considered as up-regulated, while those in blue triangles pointing downwards are considered as down-regulated.

The identification of proteins whose expression level was altered by nano-TiO_2_ treatment was performed using MALDI-TOF MS and MS/MS. No significant differences in these expressions were observed according to the type of nanoparticles. We thus choose to analyze the results obtained with the four nanoparticles as a whole. This lead to the identification of two different groups of proteins. The first group consists in proteins which are down-regulated in the presence of nano-TiO_2_ in contrast with the major part of other proteins that seem to remain unaffected. These proteins are depicted in blue triangles pointing downwards in Fig 5 and are essentially structural proteins. The second group consists in up-regulated proteins, displayed as red triangles pointing upwards in Fig 5. The proteins that differ most significantly from the bulk proteins in Fig 5 are listed in Table 1.

**Table 1:** Differentially expressed *E*. *coli* cellular proteins in response to treatment with titanium dioxide nanoparticles. Code numbers correspond to the names of those proteins whose score significantly decreases or increases in response to the treatment of the bacterial cells with TiO_2_ nanoparticles. These proteins fall into two categories, those considered as significantly down-regulated (d) and those significantly up-regulated (u) in the presence of nanoparticles.

Category	Code Number	Protein name	Bacterial strain name	Mass of protein (kDa)	Accession number	Role
d	1	chaperonin 1 (GroEL)	*E*. *coli* O1:K1	60	A1AJ51	Protein synthesis
d	2	Peptidoglycan-associated lipoprotein	*E*. *coli* O157:H7	18.8	P0A913	Membrane structure
d	3	Chaperone protein DnaK	*E*. *coli* O139:H28	68.5	A7ZHA4	Protein synthesis
d	4	Outer membrane protein C	*E*. *coli* K12	40.3	P06996	Membrane structure
d	5	Outer membrane lipoprotein slyB	*E*. *coli* O157:H7	15.6	P0A906	Membrane structure
d	6	D-galactose-binding periplasmic protein	*E*. *coli* O6	35.7	P0AEE6	Sugar metabolism
d	7	DNA protection during starvation protein	*E*. *coli* O139:H28	18.7	A7ZJM7	DNA protection against oxidative stress
d	8	50S ribosomal protein L17	*E*. *coli* O139:H28	14.4	A7ZSI3	Protein synthesis
d	9	30S ribosomal protein S6	*E*. *coli* O139:H28	15.2	P02358	Protein synthesis
d	10	Acriflavine A	*E*. *coli* O157:H7	42.2	P0AE07	Multidrug efflux system
d	11	Transaldolase B	*E*. *coli* O157:H7	35.2	P0A871	Sugar metabolism
d	17	Major outer membrane lipoprotein	*E*. *coli* O157:H7	8.3	P69778	Membrane structure
u	68	Isocitrate dehydrogenase [NADP]	*E*. *coli* K12	45.7	P08200	TCA cycle
u	99	50S ribosomal protein L5	*E*. *coli* O139:H28	20.3	A7ZSJ7	Protein synthesis
u	103	Dihydrolipoyllysine-residue acetyltransferase component of pyruvate dehydrogenase complex	*E*. *coli* K12	66.1	P06959	TCA cycle
u	105	Dihydrolipoyllysine-residue succinyltransferase component of 2-oxoglutarate dehydrogenase complex	*E*. *coli* O157:H7	44	P0AFG7	TCA cycle
u	113	30S ribosomal protein S1	*E*. *coli* O157:H7	61.1	P0AG69	Protein synthesis
u	122	Chaperone protein skp	*E*. *coli* O157:H7	17.7	P0AEU9	Protein synthesis
u	125	Elongation factor Tu 1	*E*. *coli* O139:H28	43.3	A7ZSL4	Protein synthesis
u	127	Glutamine-binding periplasmic protein	*E*. *coli* O157:H7	27.2	P0AEQ5	Amino Acid Metabolism
u	129	Glyceraldehyde-3-phosphate dehydrogenase A	*E*. *coli* O157:H7	35.5	P0A9B4	Sugar metabolism
u	135	Trigger factor	*E*. *coli* O139:H28	48.1	A7ZIJ4	Protein export
u	136	Periplasmic beta-glucosidase	*E*. *coli* K12	83.4	P33363	Sugar metabolism
u	137	Elongation factor G	*E*. *coli* O139:H28	77.5	A7ZSL5	Protein synthesis
u	138	Succinate dehydrogenase flavoprotein subunit	*E*. *coli* O157:H7	64.4	P0AC43	TCA cycle
u	139	50S ribosomal protein L2	*E*. *coli* O139:H28	29.8	A7ZSK6	Protein synthesis
u	140	Serine hydroxymethyltransferase	*E*. *coli* O139:H28	45.3	A7ZPZ4	Protein synthesis
u	141	Prolyl-tRNA synthetase	*E*. *coli* K12	63.7	C4ZRT7	Protein synthesis
u	142	ATP synthase subunit alpha	*E*. *coli* O139:H28	55.2	A7ZTU6	Protein synthesis
u	143	Tryptophanase	*E*. *coli* O139:H28	52.7	A7ZTR3	Protein synthesis
u	144	chaperonin 2 (GroES)	*E*. *coli* O139:H28	10.4	A7ZV11	Protein synthesis
u	145	50S ribosomal protein L3	*E*. *coli* O139:H28	22.2	A7ZSK9	Protein synthesis
u	146	Succinyl-CoA ligase [ADP-forming] subunit beta	*E*. *coli* O139:H28	41.4	A7ZJA8	TCA cycle
u	147	50S ribosomal protein L21	*E*. *coli* O139:H28	11.6	A7ZS83	Protein synthesis
u	148	50S ribosomal protein L4	*E*. *coli* O139:H28	22.1	A7ZSK8	Protein synthesis
u	149	Aconitate hydratase 2	*E*. *coli* K12	93.4	P36683	TCA cycle

### Results of total ATP assay

**Fig 6:**
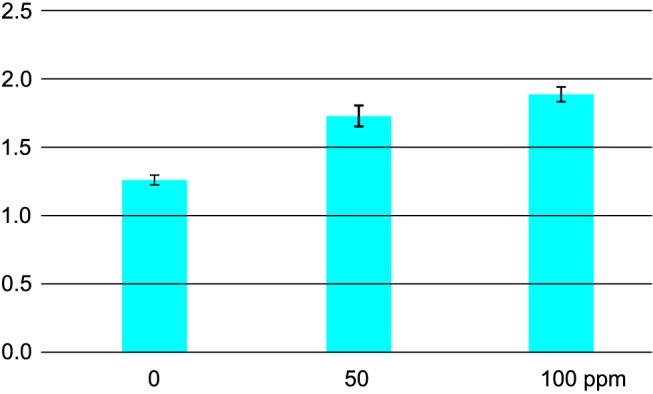
Evolution of the ATP content of a culture of *E*. *coli* (OD_600_ = 0.2) incubated for 3 h in Seine river water under room light as a function of P25 concentration.

Adenosine-5'-triphosphate (ATP), often called the "molecular unit of currency" of intracellular energy transfer, transports chemical energy within the cells for metabolism. ATP is found in and around living cells. It gives a direct measure of biological concentration or health. Fig 6 shows that the amount of “total” ATP in the medium gradually rises with P25 concentration. In a previous study [12], we have shown that bacterial survival decreases while nano-TiO_2_ concentration increases (S5 Fig). Consequently our results indicate that nano-TiO_2_ triggers a significant increase of ATP content in the medium.

## Discussion

The aim of the present study is to investigate the global metabolomic and proteomic responses of *E*. *coli* to nano-TiO_2_ stress by coupling NMR-based metabolomic and MS-based proteomic strategies. It was essential to take into account that the bacteria submitted to nano-TiO_2_ were already stressed by the transition between the rich medium in which they were grown and the Seine River water (devoid of any carbon source) where they were transferred for the need of the experiment. As the transition occurred just in the middle of the exponential phase, the bacterial cells underwent metabolic rearrangements leading to a decrease in their content of the TCA cycle metabolites [39].

Our study compared modifications in *E*. *coli* extracts incubated in presence of four different types of nano-TiO_2_, under room light (without UV) or under UV irradiation. An optimization of metabolite extraction protocol (Fig 1) allowed the successful identification of several metabolites potentially associated with nano-TiO_2_ stress (Fig 2). The photocatalytic production of ROS by nano-TiO_2_ accounts for the fact that UV_A_ irradiation leads to a difference whether cells are exposed or not to UV_A_.

The presence of nano-TiO_2_ during the incubation process of *E*. *coli* generates distinct metabolomic profiles. This clearly appears on the PCA results: the first dimension PC1 distinguishes the bacterial metabolome obtained with and without nanoparticles (see left and right distribution along the PC1 axis (Fig 3). This is clear evidence that TiO_2_ nanoparticles have an incontestable effect on bacterial metabolism.

The metabolites that carry the energy within the cell under the form of the nucleoside triphosphates (ATP, GTP, CTP and UTP) play a central role in metabolism. Their variation is clearly mediated by the presence of the nano-TiO_2_ (Fig 4). Based on the PCA analysis, it is also evident that free amino acids are released by the cells as their concentration increases under the nano-TiO_2_ treatment. This tendency is not as clear for succinate as it is for the other assigned organic acids (acetate, oxaloacetate and lactate). These four organic acids are involved in the Tricarboxylic Acid Cycle (TCA). They act as intermediates in many metabolic reactions. Two of them, acetate and oxaloacetate, are precursor of amino acids. The fatty acids are represented by signals 172 and 173. Their concentration tends also to increase under the nano-TiO_2_ treatment. These molecules could be used by cells as fuel molecules to synthesize acetate and allow neoglucogenesis which, in condition of starvation, allow bacterial cells to synthesize glucose from fatty acids.

Among the spots clustered in the "Other metabolites" panel of Fig 4, the putrescine (main component of signals 21, 107 and 142) was increased by the nano-TiO_2_ treatment. Putrescine is a catabolite of ornithine (signals 106, 124, 126 and 127, although not fully distinguishable from the signal of the acetate) and an intermediate for the synthesis of succinate (signal 113) which is an important acid involved in the TCA cycle [40]. The incubation under UV seems to tighten the link between nano-TiO_2_ presence and up-regulation of this metabolite. Putrescine, is a very common polyamine in bacteria. This molecule plays an important role during growth but is also important in acid resistance and free radical ion scavenging [41]. The increase of this metabolite could also be related to its role in the protection of the cell toward ROS because this metabolite as well as other polyamines such as spermine and spermidine play an important role in the control of the oxidative damages [42].

Proteomic brings additional information to metabolomic analysis. The nature of down-regulated and up-regulated proteins are successively discussed in terms of their involvement in the global bacterial metabolism.

Interestingly, proteomic investigations revealed that the bacterial population reacted on one hand by globally down-regulating a lot of proteins, mostly those associated with membrane integrity. On the other hand, the proteins associated with energy metabolism, including sugar metabolism and especially the TCA cycle are up-regulated. Moreover, pathways playing a direct role in biosynthetic processes such as the synthesis of amino acids or translation were modified (Fig 5).

### The down-regulated proteins

The protein most down-regulated by the 10 ppm nano-TiO_2_ treatment was the 60 kDa chaperonin whose function is the prevention of the misfolding of proteins and the promotion of the refolding and proper assembly of unfolded polypeptides generated under stress conditions [43]. These chaperonin and especially the chaperonin “groES” are the principal actors operating both for the protection of proteins from denaturation and the assembly of newly synthesized proteins. During the heat stress response *via* nickel oxide nanoparticles, 10 kDa chaperonin (grosES) proteins were detected using MALDI–TOF MS [44]. However, these proteins were not detected in our study. Peptidoglycan-associated lipoprotein (PAL) was also very much affected by contact with the four nano-TiO_2_. This protein is anchored in the outer membrane of gram negative bacteria and interacts with Tol proteins [45]. Tol proteins are involved in outer membrane stability [46, 47], while the PAL protein is required for the survival of the bacterial cells and plays an important role in pathogenesis and virulence. Its absence is responsible for an acceleration of the disorganization process of the cell envelope by nano-TiO_2_. The prokaryotic outer cell membrane is a selectively permeable membrane, which separates the periplasm from the cell surroundings. Outer membrane proteins in Gram-negative bacteria are known to be the channels for nutrients but also for drugs crossing the bacterial outer membrane [48]. Chaperone protein DnaK plays a fundamental role in normal cellular growth and maintenance [49] as in the proteostasis of *E*. *coli* [50]. The Hsp70 protein expression is greatly increased when *E*. *coli* is stressed by a variety of factors, including heat shock. This protein exhibits a slow ATPase activity and is down-regulated below detectable limit under the nano-TiO_2_. Without this protein, the cell is not able to maintain the folding and repair processes of proteins. Outer membrane protein C is also greatly affected, especially with P25 (100 ppm), which was the most toxic nanoparticle we tested [12]. This result agrees with those of Carré *et al*. who found that the main target of nano-TiO_2_ was the outer membrane proteins [51]. Another outer membrane protein, slyB decreases drastically in our conditions and under nano-TiO_2_ treatment. This protein is a small lipoprotein highly conserved in different Gram-negative bacteria that contributes to bacterial membrane integrity [52]. The significant down-regulation of this protein results in a dramatic disorganization of the outer membrane structure. Other proteins associated with transport of sugars as well as D-galactose-binding periplasmic protein were affected by nano-TiO_2_ treatment. This protein seems to be very sensitive to all concentrations of nano-TiO_2_. This sensitivity may be related to the high value of the association rate constant for sugar binding, near 10^7^ M^-1^.s^-1^ [53]. Even if the transport function of D-galactose-binding periplasmic protein is essential to the survival of bacteria, this protein acts also as a chaperone. This protein was indeed found to play an important role by increasing the refolding of unfolded proteins, protecting proteins against thermal denaturation, and forming complexes with unfolded proteins. This periplasmic substrate-binding protein act in a manner similar to that of molecular chaperones [54].

“DNA protection during starvation protein” is a protein that binds the chromosome non-specifically, forming a highly ordered and stable DNA-binding proteins from starved cells (dps)-DNA co-crystal within which chromosomal DNA is condensed and protected from diverse damages. This protein is essentially expressed during the stationary phase. Under nano-TiO_2_ treatment, its function seems to be lost because its concentration dramatically decreased, exposing DNA to free iron and consequently to ROS formed during the Fenton reaction and nano-TiO_2_ attack [55]. In addition to other proteins, 30S S6 and 50S L17 ribosomal proteins were also down-regulated. Usually, ribosomal proteins are involved in protein turn over and biosynthesis but their down-regulation as a result of nano-TiO_2_ treatment is very difficult to interpret. There are more than twenty ribosomal proteins and their assembly is strictly regulated. The ribosomal protein S6 has a special function in the cell as it can be phosphorylated and dephosphorylated in response to numerous cell disorders [56]. Its decrease confirms its involvement in the loss of cellular integrity leading to death [57] but its increase was also observed as a result of nano-TiO_2_ treatment (see below). Acriflavine resistance protein A (AcrA) is another very important protein whose fundamental physiological function is, like the acriflavine resistance protein B, to protect *E*. *coli* against naturally occurring hydrophobic inhibitors such as biliary salts and fatty acids, which are present in the natural environment of this enteric bacteria. However, usually, the increase of the transcription of acrAB is associated to general stress response [58]. But, in our conditions, we observed the decrease of the content of this protein, especially in response to low concentrations of nano-TiO_2_ (10 ppm). Finally, the virtual disappearance of the lipoprotein “LPP” in presence of 100 ppm of all tested nano-TiO_2_ was very significant: LPP is the most abundant protein in *E*. *coli*, and it amounts to more than 500,000 molecules per cell. This major outer LPP interacts with the peptidoglycan both covalently and non-covalently [59]. The lipoprotein “LPP” normally cross-links the bacterial outer membrane to the peptidoglycan lattice. This interaction contributes to the maintenance of the cellular integrity and is responsible for the death of the cells. This may appear only at high nano-TiO_2_ concentration, 100 ppm, since at the lower concentration of 10 ppm, no significant decrease can be noticed.

In short, our proteomic data shows that the proteins most affected by the exposure to nanoparticles are the one associated with the integrity of the membrane, like outer-membrane proteins, proteins involved in the proteostasis such as ribosomal proteins, proteins involved in the heat shock or in the stress response, proteins involved in the DNA protection during starvation, or proteins which promote protein folding. This corroborates metabolomic results which show the presence of large amount of amino acids produced by proteolysis, suggesting that lower amounts of proteins could be due to their degradation by proteases.

### The up-regulated proteins

Whereas down-regulated protein are mainly external proteins in direct contact with nanoparticles, up-regulated proteins are part of the inner part of the bacteria. The up-regulated proteins belong to the energy metabolism, especially glycolysis and the TCA cycle. They are found in Fig 5 (upper right) and are highlighted in red.

Glyceraldehyde-3-P dehydrogenase, one of the first enzymes of the glycolysis, is up-regulated by nano-TiO_2_. The glyceraldehyde-3-P dehydrogenase is involved in the oxidation of the glyceraldehyde 3P into 1,3-diPhosphoglycerate before the formation of ATP. The dihydrolipoyl lysine-residue acetyltransferase component of pyruvate dehydrogenase is also found to be one of the up-regulated enzymes most expressed under 100 ppm nano-TiO_2_ treatment. This enzyme belongs to the last of the 3 compounds of the complex pyruvate dehydrogenase (PdH). This is the last step of glycolysis and it drives the synthesis of acetyl-Coenzyme A. This coenzyme enters the TCA cycle and this step is fundamental for the energy metabolism of the bacteria. Succinyl-CoA ligase, also named succinyl-CoA synthetase (ADP-forming), is the most up-regulated detected protein at 100 ppm of nano-TiO_2_. It is an important protein, which catalyzes, in the TCA cycle, the ADP-synthesis dependent ligation of succinate and CoA to form succinyl-CoA. However, the succinyl-CoA ligase is also reversible and here we observe the up-regulation of the enzyme that forms succinate. This corroborates metabolomic results showing, that, in the presence of nano-TiO_2_, the concentration of oxaloacetate increases due to the transformation of succinate in the TCA cycle. Because this reaction is reversible, increase or decrease of the activity of this enzyme should depend on the ATP/ADP ratio [60]. Under normal conditions, the cells produce reduced nucleotides through the pentose phosphate and glycolytic pathways, as well as TCA cycle. Then, the cells obtain ATP either through the glycolytic pathway with acetate synthesis or through the electron transport chain with oxidation of the reduced nucleotides [61]. In the absence of glucose, the cells can use acetate with the shutdown of the glycolysis, the activation of the acetate utilization pathways and the activation of neoglucogenesis [39, 62, 63].

Aconitate hydratase (aconitase) is the most up-regulated protein by the 10 ppm nano-TiO_2_ treatment. This enzyme, which drives the isocitrate formation, appears to be one of the first enzymes of the TCA cycle. But aconitases have dual functions, as enzymes in the TCA cycle and as regulators of iron metabolism [64]. Aconitase also regulates the protein amount of several genes that are involved in cell division like ftsZ, and in DNA repair like recA and uvrB, allowing the cell to quickly adapt to the variation of the environmental conditions [65]. Succinate dehydrogenase is also up-regulated by the 10 ppm nano-TiO_2_ treatment. This is the only enzyme that is involved in both the TCA cycle and the electron transport chain [65]. The succinate dehydrogenase plays an important role in the transformation of succinate into fumarate. This enzyme is regarded as an important enzyme of the TCA cycle. Isocitrate dehydrogenase catalyzes the oxidative decarboxylation of isocitrate, producing 2-oxoglutarate and reducing NAD+ to NADH [66]. This enzyme is up-regulated under 100 ppm nano-TiO_2_ treatment.

Another important protein whose production increases under nano-TiO_2_ treatment, particularly with 100 ppm nano-TiO_2_, is the dihydrolipoyl-lysine residue succinyltransferase component of 2-oxoglutarate dehydrogenase complex that catalyzes the overall conversion of 2-oxoglutarate to succinyl-CoA and CO_2_ [67]. Its production is dramatically increased under 100 ppm nano-TiO_2_ showing that amino acid degradation is promoted by a huge concentration of nano-TiO_2_. It is well known that four amino acids promote succinyl-coA synthesis. Among them tryptophan is a glucogenic amino acid. Thus, the degradation of tryptophan may represent a possible pathway for bacteria to save energy. Accordingly, the tryptophanase production is also increased under nano-TiO_2_ treatments. This protein is a pyridoxal phosphate-dependent enzyme responsible for the production of indole, an important intra- and interspecies signaling molecule in bacteria [68]. Indole is a stable biological compound. *E*. *coli* is known to utilize indole to protect itself against other microorganisms [69]. Consequently, the nano-TiO_2_ induces the increase of indole production by the bacterial population. One hypothesis is that bacteria could utilize indole they produce to protect themselves from the stress induced by nano-TiO_2_. Unfortunately, the signals of indole are not distinguishable on the NMR spectra.

Another protein whose production is involved in the cell protein turn-over is the elongation factor TU (EF-TU). The production of this protein increases significantly under 100 ppm nano-TiO_2_ treatment. This elongation factor is an important component of the multistep ribosomal decoding pathway that ensures rapid and accurate translation [70]. This is the carrier of aminoacyl-tRNA essential in protein biosynthesis [71]. Its increase confirms that, in our conditions, a sub-population of bacteria was trying to synthesize new proteins in order to survive to nano-TiO_2_ treatment. Under rapid growth conditions, the translation elongation factor EF-TU is the most abundant protein in most bacterial cells, reaching ≈10 times the concentration of ribosomes [72].

In parallel to the increase in production of proteins involved in the catabolism of amino acids such as tryptophanase, we observed the up-regulation of 5 ribosomal proteins: the 50S ribosomal proteins L2, L3, L4, L5 and L21. The increase of these proteins highlights the important role of protein turn-over in the maintenance of cell fitness as a response to nano-TiO_2_ treatment [73].

Overall, our proteomic data show that the proteins which are the most up-regulated are those associated to the energy metabolism such as glycolysis, TCA cycle and proteins involved in the electron transport chain. These results reinforce the metabolomic data which shows an increase of amino acids, suggesting that the lower amounts of the proteins could be due to their degradation by proteases.

Thanks to the use of MALDI-TOF MS and MS/MS methods, we detected a larger number of proteins affected by nano-TiO_2_ than Carré et al. [51] did, when they studied the effect of nano-TiO_2_ P25 with or without UV_A_ on the proteome of *E*. *coli* ATCC 8739. They did observe a degradation of some proteins, proportional to the concentration of the nano-TiO_2_. In their experimental conditions, Carré et al. found between 7 and 22 different proteins whose number decreased under nano-TiO_2_ treatment and they asserted that the targeted proteins were very heterogeneous in cellular localization and belonged to different functional families. Here, we detected much more proteins affected by the nano-TiO_2_ stress. These proteins cluster into two major groups: those involved in stress response and those involved in energy metabolism. Among the common proteins observed both by Carré et al. [51] and our team are the chaperone DnaK and the 30S ribosomal S6. In addition to the proteins detected by Carré et al., we have found a down-regulation of proteins likely directly in contact with the nano-TiO_2_. It includes the outer membrane proteins A, C, the lipoprotein slyB, or the lipoprotein associated with the peptidoglycan. However, we found that other proteins, which are involved in the normal cell growth, like the chaperonin 1, were also affected by the nano-TiO_2_ treatment.

Since so far "omics" analyses reflect a statistical average of the state of a large number of bacteria, it cannot give clues on its distribution in the bacterial population. Our proteomic results cannot rule out that the bacteria may not react in the same way to the presence of nano-TiO_2_ when one part of the population is able to adapt to the nano-TiO_2_. Indeed, the moderate toxicity observed (from 20% mortality for 100 ppm R nano-TiO_2_ to 80% for P25 nano-TiO_2_) (S4 Fig) can be accounted for by heterogeneity in cell—nano-TiO_2_ interactions. In our previous study, TEM and SEM imaging coupled with surface charge measurements have demonstrated the presence of two populations of bacteria. One part of the population strongly interacts with nano-TiO_2_ (strong adsorption of nano-TiO_2_ on cell surface) while the other is totally deprived of nano-TiO_2_ on the cell surface (S5 Fig). The heterogeneity of response to nanoparticles may come from either a different physiological and/or metabolomic state or from genetic variability, although the latter hypothesis is less probable.

Recently, Sohm et al. [74] confirmed that the nano-TiO_2_ toxicity was due to the adsorption of the nano-TiO_2_ on the cell surface, resulting in membrane destabilization, triggering the collapse of membrane potential and higher membrane permeability with consequently the inhibition of the ATPase, but also possibly the release of ATP outside the cell. Along the same line, Cui et al. [75], found that gold nanoparticles exert their antibacterial activities mainly by two ways: one is to collapse membrane potential, inhibiting ATPase activities; the other is to inhibit the subunit of the ribosome from binding tRNA [75]. We have not determined whether the ATPase is affected, but all our data points towards a dramatic decrease of the ATP level inside the cell correlated with an increased ATP released in the medium (Fig 6). Here, we found a huge concentration of ATP in the medium with up to 2 mM for 100 ppm of TiO_2_, while the usual extracellular concentration is about to 3 to 5% that of the intracellular one, which is about 1 to 5 mM [76]. Extracellular ATP may originate from cell death or compromised membranes. Consequently, this suggests that the ATP released by some members of the bacterial communities may supply energy to other members and hence help the communities survive, as demonstrated previously [76]. Previously, we observed that nano-TiO_2_ treatment on *E*. *coli* [12] induces a global decrease of bacterial survival during nano-TiO_2_ treatment (S4 Fig). Moreover, in Seine River medium, the cells are exposed to starvation, leading to cell death. However, the abundance of ribosomal proteins and growth markers indicates a stimulation of biosynthetic pathways and a non-inhibited growth of some *E*. *coli* cells. The proteomic and metabolomic analyses suggest that energetic needs of surviving cells may be met by an increase of both glycolysis and TCA cycle. We propose thus that the major part of bacteria population is dying in response to the presence of nano-TiO_2,_ as evidenced by the increase of ATP in the medium helping the other to survive. On the other hand, the presence of some up-regulated proteins (succinate dehydrogenase [77], aconitate hydratase [20,78]) and metabolites (putrescine [42], valine [79]) shows the resistance of the other part of the population. These last proteins and especially aconitate hydratase have been identified to play a role against oxidative stress in addition to their principal functions [80]. These proteins could represent a signature of the minor surviving population.

## Conclusion

Combined NMR-based metabolomics and MS-based proteomics are powerful tools that provides valuable mechanistic insights into the effects of chemical and other environmental stressors (osmotic, starvation or temperature stress) [39], and are emerging as valuable tools for assessing the effects of chemical pollutants on organisms. To deal with biological variability, the analysis of large samples is required but several computational tools are available to minimize the impact of unwanted variance, such as unsupervised multivariate methods (PCA) and data filtering techniques. We employed a coupled metabolomic and proteomic approach to help elucidating the mode of low antibacterial action of nano-TiO_2_ toward *E*. *coli* in natural water. Results from our proteomic analyses, confirmed by metabolomic ones, revealed that exposure of *E*. *coli* cells to nano-TiO_2_ resulted in heterogeneous bacterial responses. One part of the population is able to adapt to this stress and temporarily survive; another part cannot adapt to this stress and dies progressively. A mechanism of oxidative stress is proposed regarding the capacity of nano-TiO_2_ to generate ROS as it was shown (S6 Fig, unpublished results) and some up-regulated proteins are involved in oxidative stress protection. The absence of interaction between nano-TiO_2_ and the rest of the bacterial population, while some bacteria are fully covered by nano-TiO_2_, was highlighted in our previous study and accounts for discrepancies in reaction when faced with nano-TiO_2_. The use of combined proteomics and metabolomics may be a breakthrough to precisely evaluate the level of toxicity of nano-TiO_2_ under environmental conditions. This heterogeneous behavior of bacteria facing nanoparticles with moderate oxidative stress capacity in natural environment may correspond to a sacrificial collective behavior. The driving force for this appealing effect, the type of contaminants and exposure conditions remain a significant problem to address for nano-ecotoxicity studies. In addition, this study brings a complete description of the toxicity of metallic engineered nanoparticles, considering as a whole the environmental medium, the bacterial fitness in response to nanoparticles and the physico-chemical characterization of these nanomaterial. These experiments show that some proteins and metabolites are potentially associated with nano-TiO_2_ stress. Among them, the best biomarkers might be chaperonin 1 and isocitrate dehydrogenase as their amount decrease and increase significantly in the presence of nano-TiO_2_, respectively. Some metabolites might also be useful biomarkers, for example phenylalanine or valine. The combined assays of some proteins and metabolites biomarkers constitute probably the best witness of the toxicity of nano-TiO_2_ natural waters.

## Supporting information

S1 Fig An example of region definition and baseline correction method for integration. Part of the spectrum of Fig 2 of main text, showing region of integration 17 (see S2 Table).
(PDF)

S2 Fig Comparison of the upfield part of NMR ^1^H spectra of *E*. *coli* MG1655 metabolites as a function of the boiling time. Downfield part in Fig 1A of the main text.
(PDF)

S3 Fig Comparison of the upfield part of NMR ^1^H spectra of *E*. *coli* MG1655 metabolites as a function of perchloric acid concentration. Downfield part in Fig 1B of the main text.
(PDF)

S4 Fig Toxicity assessment of nano-TiO_2_ toward *E*. *coli*. Survival (normalized to control without nanoparticles) of *E*. *coli*, incubated under room light during 3h at 37°C in Seine river water with increasing concentrations of nano-TiO_2_: P25 (red), R (blue), M (green), A (purple).
(PDF)

S5 Fig TEM images of *E*. *coli* in contact with R type nano-TiO_2_. 1000ppm of R (up) or 100 ppm (down).
(PDF)

S6 Fig Evolution hydroxyl and superoxide radicals produced by photocatalysis of P25 and R TiO_2_ nanoparticles. (PDF)

S1 Table Physicochemical characterization of the TiO_2_ nanoparticles used in this study. (PDF)

S2 Table Compiled data for ^1^H NMR spectrum of Fig 2. For each area: number, maximum and minimum chemical shift, letter for the assignment on Fig 2, assignment, association of metabolite to their class. Classes: NNA: nucleosides, nucleotides and analogues; AA: amino acids; CAH: carbohydrates; OA: organic acids; OM: other metabolites; SDC: superimposition of different classes.
(PDF)
