# Supplementary material for: Metabolomic and proteomic investigations of impacts of titanium dioxide nanoparticles on Escherichia coli
Source: PLoS One. 2017 Jun 1;12(6):e0178437. doi: 10.1371/journal.pone.0178437 (PMC5453534; doi:10.1371/journal.pone.0178437)
Supplement: S1 Fig — Part of the spectrum of Fig 2 of main text, showing region of integration 17 (see S2 Table). (PDF) [file pone.0178437.s001.pdf]

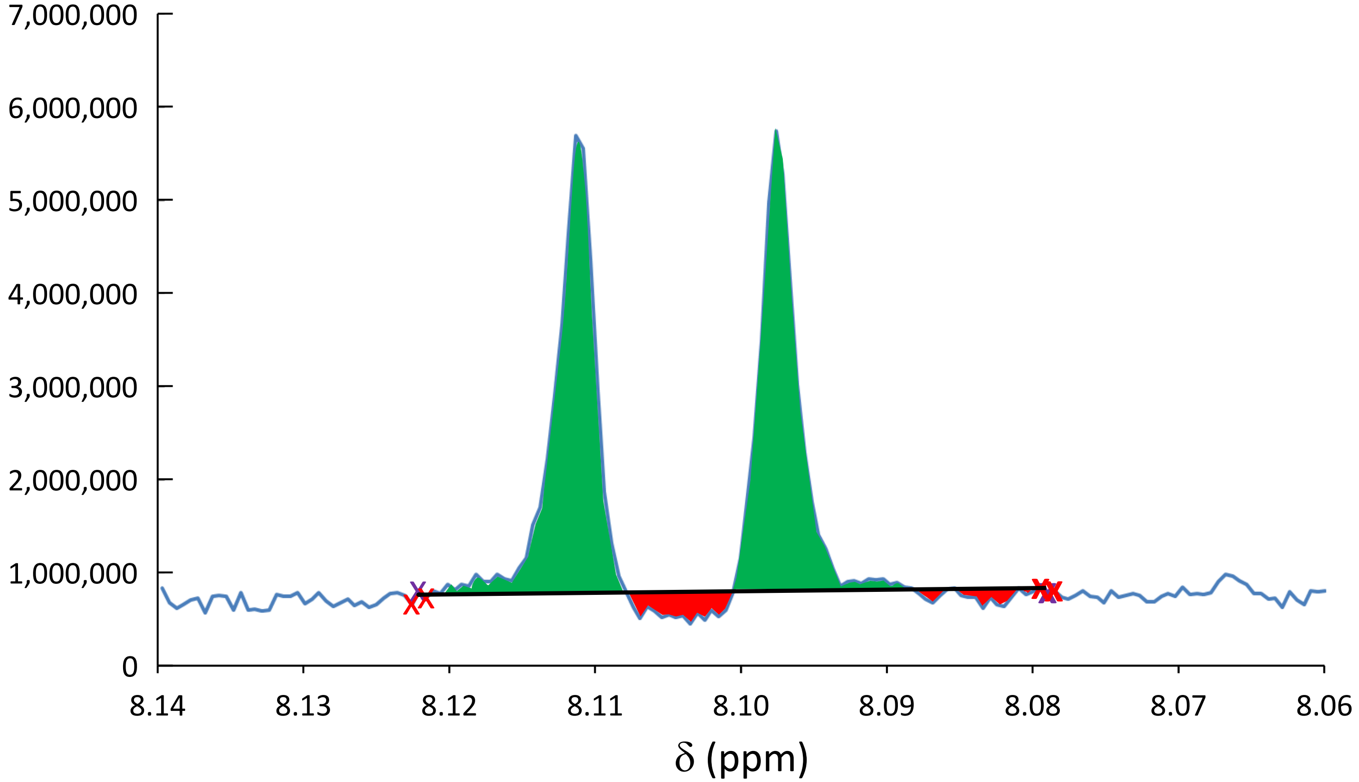

S1 Fig. An example of region definition and baseline correction method for integration.

Part of the spectrum of Fig 2 of main text, showing region of integration 17 (see S2 Table). The limit of the area is defined by the purple crosses. At each extremity, the average between the intensity at the purple cross and the intensity at the two adjacent red crosses is performed to calculate the local baseline of the signal, considering the linear variation with chemical shift. Then, integration is performed by summing point intensities between the purple crosses and subtracting the intensity of the baseline point, such that only on the green (positive) and red (negative) areas are taken into account. This method allows the integration of a sharp signal superimposed with a broad one.
