# Supplementary material for: Metabolomic and proteomic investigations of impacts of titanium dioxide nanoparticles on Escherichia coli
Source: PLoS One. 2017 Jun 1;12(6):e0178437. doi: 10.1371/journal.pone.0178437 (PMC5453534; doi:10.1371/journal.pone.0178437)
Supplement: S2 Fig — Downfield part in Fig 1A of the main text. (PDF) [file pone.0178437.s002.pdf]

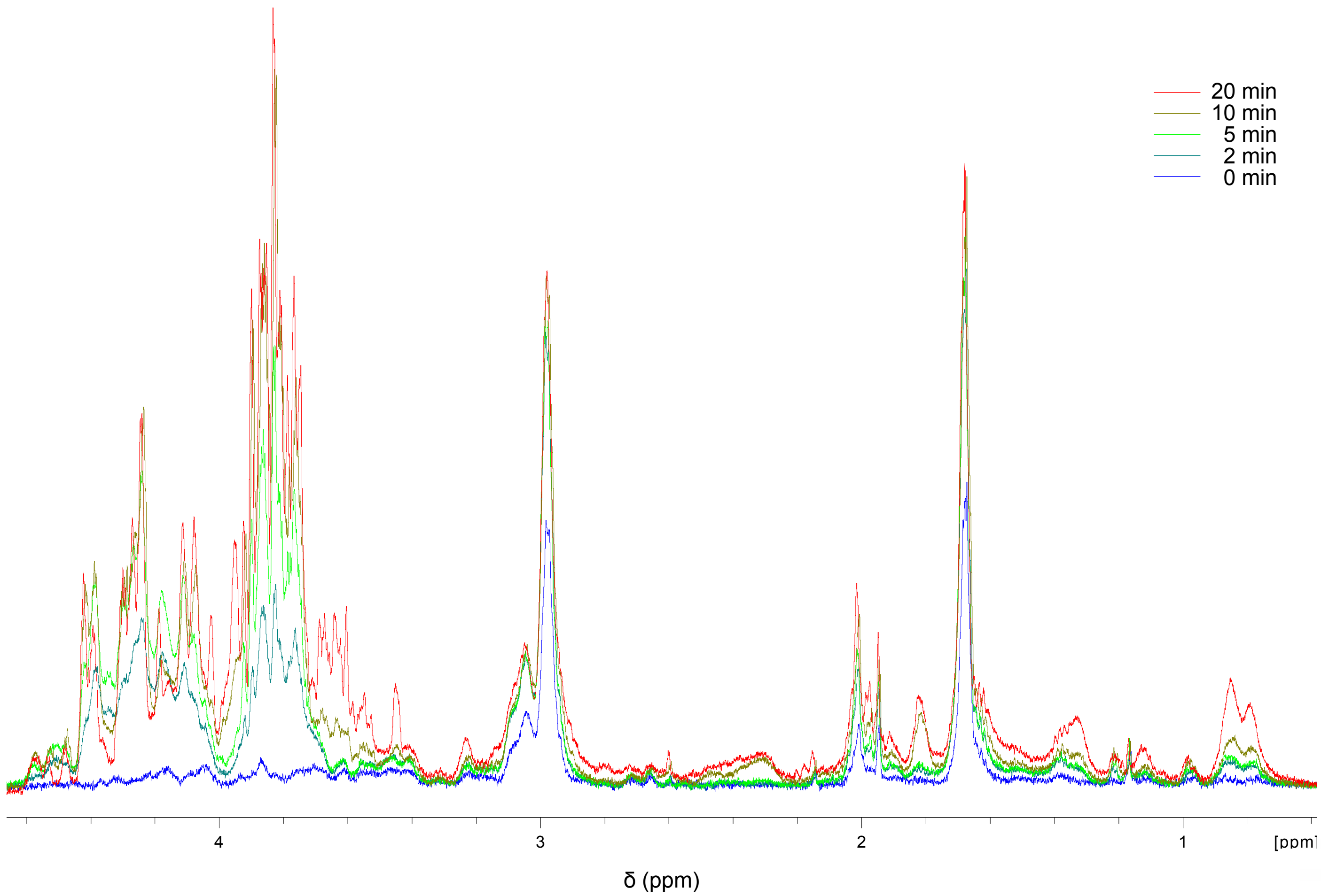

S2 Fig. Comparison of the upfield part of NMR ^{1}H spectra of *E. coli* MG1655 metabolites as a function of the boiling time.

Downfield part in Fig. 1a of the main text.
