# Supplementary material for: Metabolomic and proteomic investigations of impacts of titanium dioxide nanoparticles on Escherichia coli
Source: PLoS One. 2017 Jun 1;12(6):e0178437. doi: 10.1371/journal.pone.0178437 (PMC5453534; doi:10.1371/journal.pone.0178437)
Supplement: S3 Fig — Downfield part in Fig 1B of the main text. (PDF) [file pone.0178437.s003.pdf]

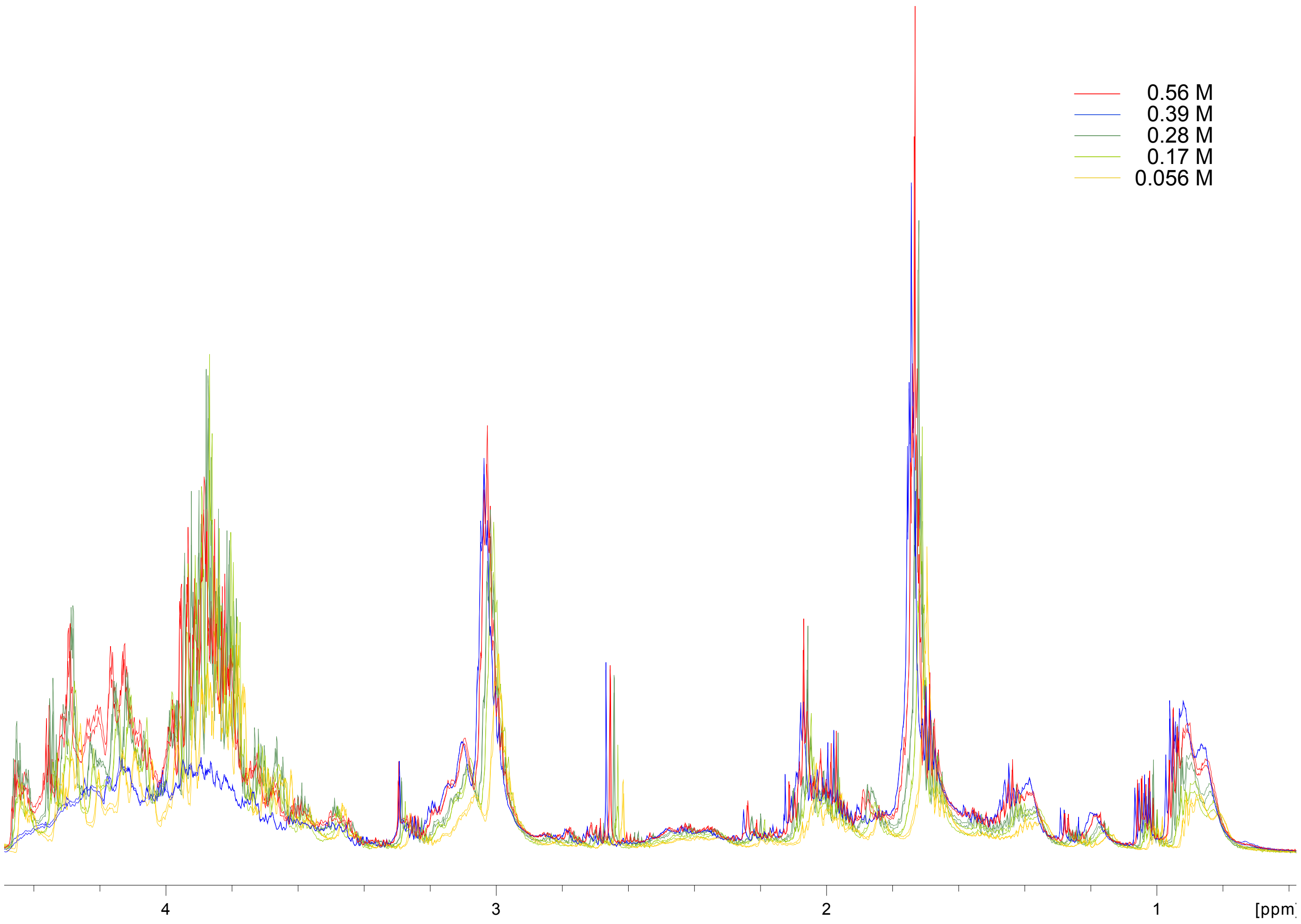


**S3 Fig. Comparison of the upfield part of NMR $^1$H spectra of *E. coli* MG1655 metabolites as a function of perchloric acid concentration.**

Downfield part in Fig. 1b of the main text.
