# Supplementary material for: Metabolomic and proteomic investigations of impacts of titanium dioxide nanoparticles on Escherichia coli
Source: PLoS One. 2017 Jun 1;12(6):e0178437. doi: 10.1371/journal.pone.0178437 (PMC5453534; doi:10.1371/journal.pone.0178437)
Supplement: S4 Fig — Survival (normalized to control without nanoparticles) of E. coli, incubated under room light during 3h at 37°C in Seine river water with increasing concentrations of nano-TiO2: P25 (red), R (blue), M (green), A (purple). (PDF) [file pone.0178437.s004.pdf]

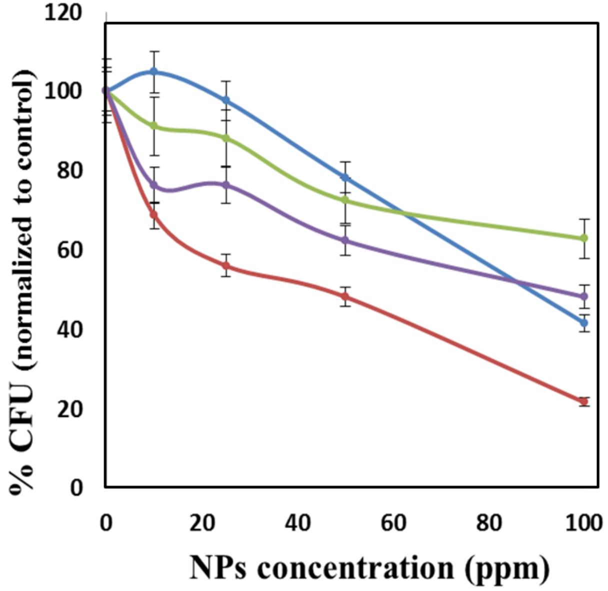


**S4 Fig. Toxicity assessment of nano-TiO2 toward *E. coli*.**

Survival (normalized to control without nanoparticles) of *E. coli*, incubated under room light during 3h at 37°C in Seine river water with increasing concentrations of nano-$TiO_2$: P25 (red), R (blue), M (green), A (purple).
