# Supplementary figures and images for: Metabolomic and proteomic investigations of impacts of titanium dioxide nanoparticles on Escherichia coli
Source: PLoS One. 2017 Jun 1;12(6):e0178437. doi: 10.1371/journal.pone.0178437 (PMC5453534; doi:10.1371/journal.pone.0178437)

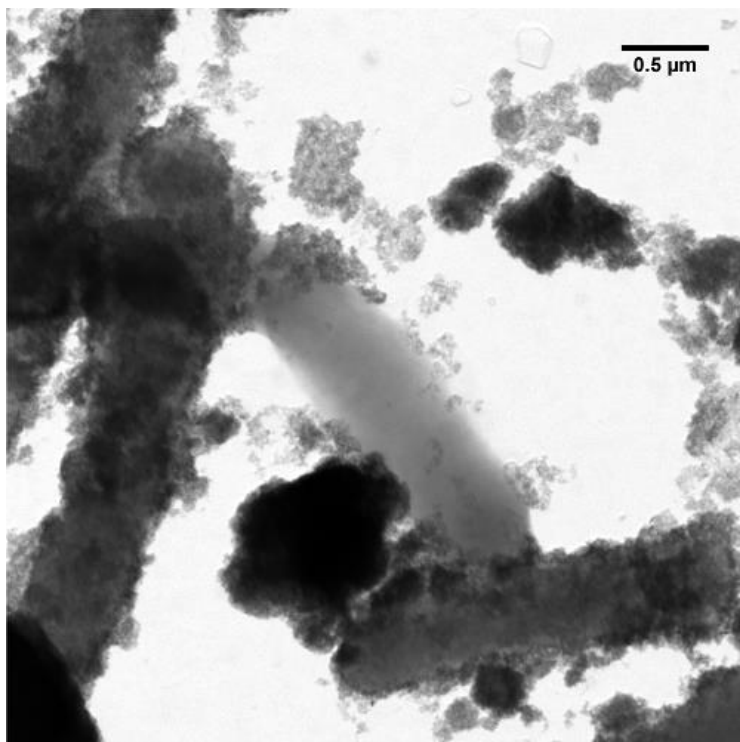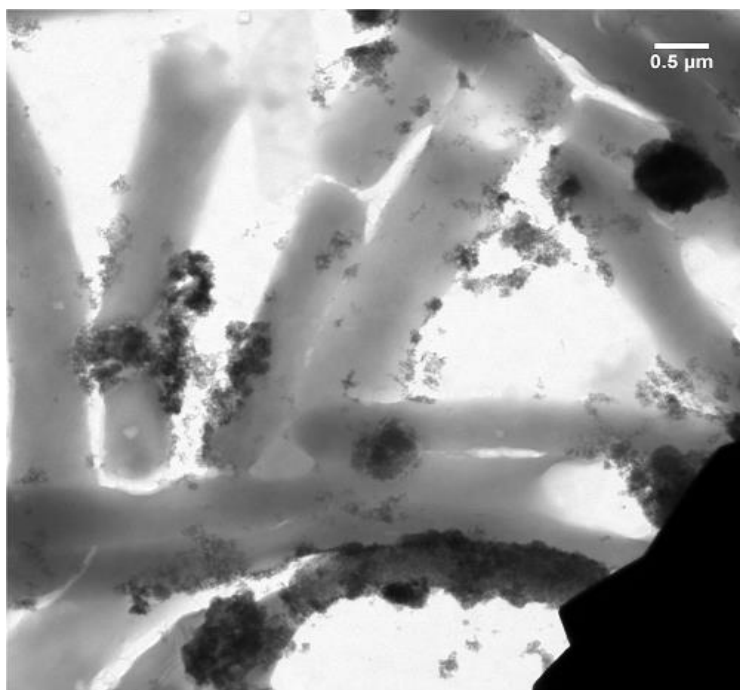

**S5 Fig. TEM images of *E. coli* in contact with R type nano-TiO<sub>2</sub>.  
1000ppm of R (up) or 100 ppm (down).**

Supplement: S5 Fig — 1000ppm of R (up) or 100 ppm (down). (PDF) [file pone.0178437.s005.pdf]
