# Supplementary material for: Metabolomic and proteomic investigations of impacts of titanium dioxide nanoparticles on Escherichia coli
Source: PLoS One. 2017 Jun 1;12(6):e0178437. doi: 10.1371/journal.pone.0178437 (PMC5453534; doi:10.1371/journal.pone.0178437)
Supplement: S6 Fig — (PDF) [file pone.0178437.s006.pdf]

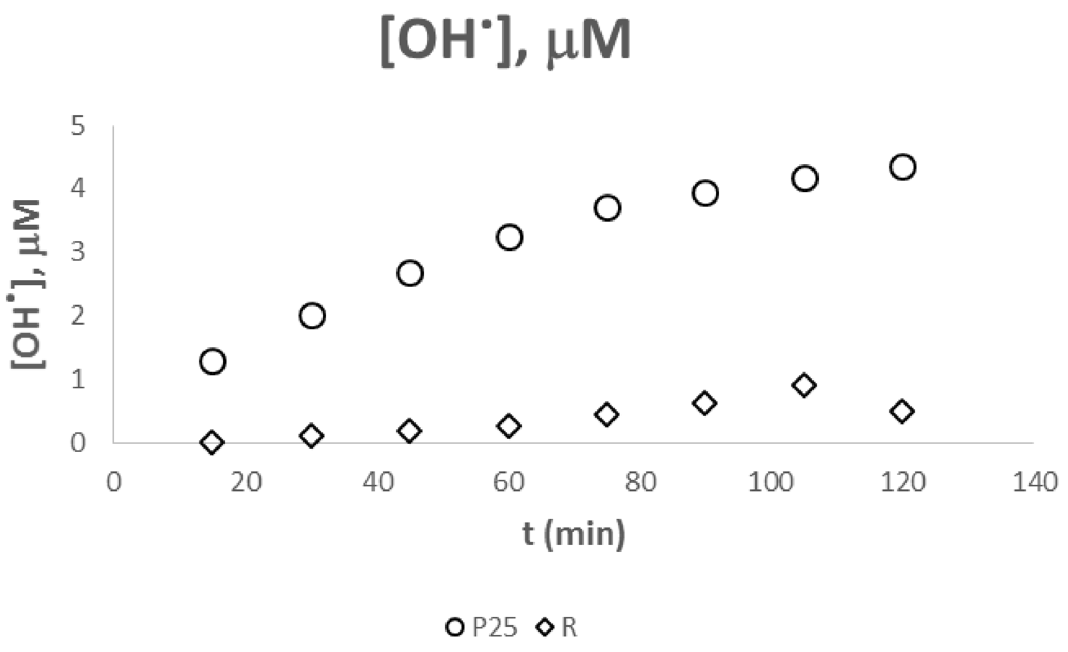

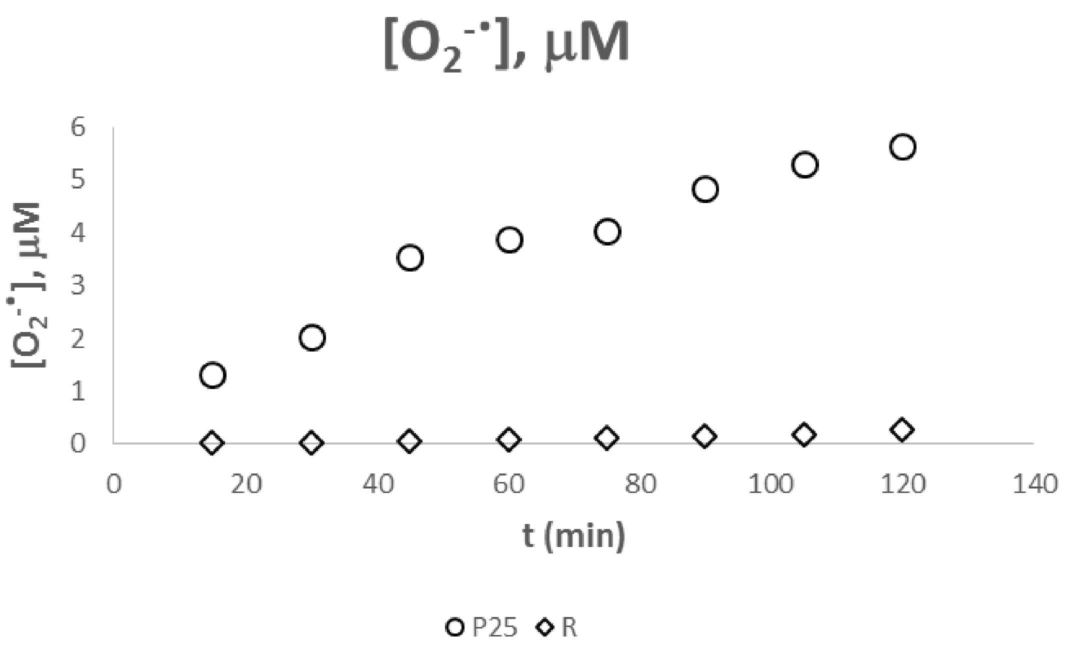

S6 Fig. – Evolution hydroxyl and superoxide radicals produced by photocatalysis of P25 and R TiO_2 nanoparticles in Seine Water River.
