# Supplementary material for: Metabolomic and proteomic investigations of impacts of titanium dioxide nanoparticles on Escherichia coli
Source: PLoS One. 2017 Jun 1;12(6):e0178437. doi: 10.1371/journal.pone.0178437 (PMC5453534; doi:10.1371/journal.pone.0178437)
Supplement: S1 Table — (PDF) [file pone.0178437.s007.pdf]

S1 Table - Physicochemical characterization of the TiO_2 nanoparticles used in this study

Sample Name	Composition (XRD)	Crystallite size (XRD)	Particle shape (TEM)	Average particle size, nm (TEM)	Surface area (SAXS)	IsoElectric point	Mean size of aggregates
R	100% rutile	17 nm	Rods	25 ± 5 x4	150 m^2/g	6.0 ± 0.1	850 nm
M	50% rutile<br>50% anatase	37 nm<br>9 nm	Rutile rods<br>Anatase spheres	20 ± 3 x3<br>8.5 ± 2	70 m^2/g	6.2 ± 0.1	360 nm
P25	21% rutile<br>79% anatase	48 nm<br>55 nm	Rutile cubes<br>Anatase spheres	37 ± 5<br>22 ± 6	50 m^2/g	6.2 ± 0.1	850 nm
A	100% anatase	8 nm	Spheres	11 ± 2	150 m^2/g	6.3 ± 0.1	660 nm
