# Supplementary material for: Metabolomic and proteomic investigations of impacts of titanium dioxide nanoparticles on Escherichia coli
Source: PLoS One. 2017 Jun 1;12(6):e0178437. doi: 10.1371/journal.pone.0178437 (PMC5453534; doi:10.1371/journal.pone.0178437)
Supplement: S2 Table — For each area: number, maximum and minimum chemical shift, letter for the assignment on Fig 2, assignment, association of metabolite to their class. Classes: NNA: nucleosides, nucleotides and analogues; AA: amino acids; CAH: carbohydrates; OA: organic acids; OM: other metabolites; SDC: superimposition of different classes. (PDF) [file pone.0178437.s008.pdf]

**S2 Table** – Compiled data for $^{1}$H NMR spectrum of Fig 2

| Region number | Max | Min | letter assignment on figure 2 | Assignment | Assigned with | Class |
|---|---|---|---|---|---|---|
| 1 | 9.42 | 9.395 | a0 | nicotinamide adenine dinucleotide phosphate | (1) TOCSY [b] | NNA |
| 2 | 9.395 | 9.362 | b | nicotinamide adenine dinucleotide | (1) TOCSY [b] | NNA |
| 3 | 9.26 | 9.212 | | | | |
| 4 | 9.212 | 9.165 | | | | |
| 5 | 9.035 | 9.01 | c | 5'-GMP | (1) TOCSY [c] | NNA |
| 6 | 9.01 | 8.988 | c | 5'-GMP | (1) TOCSY [c] | NNA |
| 7 | 8.65 | 8.634 | | | | |
| 8 | 8.57 | 8.534 | | | | |
| 9 | 8.533 | 8.509 | d | 5'-AXP | | NNA |
| 10 | 8.509 | 8.49 | d | 5'-AXP | | NNA |
| 11 | 8.487 | 8.462 | | | | |
| 12 | 8.42 | 8.397 | e | 5',3'-cAMP | (1) TOCSY [c] | NNA |
| 13 | 8.397 | 8.382 | f | 5',3'-cAMP | (1) TOCSY [c] | NNA |
| 14 | 8.361 | 8.348 | | | | |
| 15 | 8.335 | 8.3 | | | | |
| 16 | 8.184 | 8.173 | | | | |
| 17 | 8.123 | 8.08 | g | 5'-CMP or 5'-dCMP | (1) TOCSY | NNA |
| 18 | 8.047 | 8.022 | g | 5'-dCMP or 5'-CMP | (1) TOCSY | NNA |
| 19 | 7.844 | 7.815 | h | 5'-UMP or UDP-glucose | (1) | NNA |
| 20 | 7.795 | 7.769 | h | UDP-glucose or 5'-UMP | (1) | NNA |
| 21 | 7.69 | 7.398 | i | $NH_3^+$ moieties, mainly putrescine, also cadaverine and ornithine | (1) TOCSY | OM |
| 22 | 7.672 | 7.598 | | | | |
| 23 | 7.392 | 7.379 | j | phenylalanine | (1) TOCSY | AA |
| 24 | 7.379 | 7.368 | j | phenylalanine | (1) TOCSY | AA |
| 25 | 7.299 | 7.287 | j | phenylalanine | (1) TOCSY | AA |
| 26 | 7.287 | 7.276 | j | phenylalanine | (1) TOCSY | AA |
| 27 | 7.162 | 7.122 | k | ammonium | $^{14}$N coupling [d] | OM |
| 28 | 7.1 | 7.075 | | | | |
| 29 | 7.075 | 7.034 | k | ammonium | $^{14}$N coupling [d] | OM |
| 30 | 6.988 | 6.947 | k | ammonium | $^{14}$N coupling [d] | OM |
| 31 | 6.79 | 6.725 | | | | |
| 32 | 6.257 | 6.235 | l | 5'-AXP & 5',3'-cAMP | (1) TOCSY | NNA |
| 33 | 6.213 | 6.18 | m | 5'-CMP and 5'-dCMP | (1) TOCSY | NNA |
| 34 | 6.164 | 6.143 | | | | |
| 35 | 6.058 | 6.042 | | | | |
| 36 | 6.009 | 5.981 | n | 5'-GMP | (1) | NNA |

| | | | | | | |
|---|---|---|---|---|---|---|
| | | | | | TOCSY | |
| 37 | 5.914 | 5.894 | | | | |
| 38 | 5.894 | 5.877 | | | | |
| 39 | 5.869 | 5.84 | | | | |
| 40 | 5.369 | 5.356 | o | carbohydrates and fatty acids | (1) | SDC |
| 41 | 5.356 | 5.326 | o | carbohydrates and fatty acids | (1) | SDC |
| 42 | 5.175 | 5.17 | o | carbohydrates and fatty acids | (1) | SDC |
| 43 | 5.167 | 5.16 | o | carbohydrates and fatty acids | (1) | SDC |
| 44 | 5.152 | 5.146 | o | carbohydrates and fatty acids | (1) | SDC |
| 45 | 5.146 | 5.139 | o | carbohydrates and fatty acids | (1) | SDC |
| 46 | 5.139 | 5.132 | o | carbohydrates and fatty acids | (1) | SDC |
| 47 | 4.555 | 4.525 | p | nucleoside derivatives, carbohydrates and amino acids | (1) | SDC |
| 48 | 4.508 | 4.485 | p | nucleoside derivatives, carbohydrates and amino acids | (1) | SDC |
| 49 | 4.438 | 4.429 | p | nucleoside derivatives, carbohydrates and amino acids | (1) | SDC |
| 50 | 4.429 | 4.42 | p | nucleoside derivatives, carbohydrates and amino acids | (1) | SDC |
| 51 | 4.42 | 4.413 | p | nucleoside derivatives, carbohydrates and amino acids | (1) | SDC |
| 52 | 4.413 | 4.407 | p | nucleoside derivatives, carbohydrates and amino acids | (1) | SDC |
| 53 | 4.407 | 4.399 | p | nucleoside derivatives, carbohydrates and amino acids | (1) | SDC |
| 54 | 4.399 | 4.384 | p | nucleoside derivatives, carbohydrates and amino acids | (1) | SDC |
| 55 | 4.384 | 4.377 | p | nucleoside derivatives, carbohydrates and amino acids | (1) | SDC |
| 56 | 4.342 | 4.336 | q | mainly 5'-GMP | (1) TOCSY | NNA |
| 57 | 4.336 | 4.326 | q | mainly 5'-GMP | (1) TOCSY | NNA |
| 58 | 4.326 | 4.316 | q | mainly 5'-GMP | (1) TOCSY | NNA |
| 59 | 4.316 | 4.309 | q | mainly 5'-GMP | (1) TOCSY | NNA |
| 60 | 4.297 | 4.275 | r | Mainly lactate. Also nucleoside derivatives, carbohydrates and amino acids | (1) TOCSY | SDC |
| 61 | 4.275 | 4.245 | r | Mainly lactate. Also nucleoside derivatives, carbohydrates and amino acids | (1) TOCSY | SDC |
| 62 | 4.217 | 4.197 | s | nucleoside derivatives, carbohydrates and amino acids | (1) | SDC |
| 63 | 4.185 | 4.153 | s | nucleoside derivatives, carbohydrates and amino acids | (1) | SDC |
| 64 | 4.148 | 4.118 | s | nucleoside derivatives, carbohydrates and amino acids | (1) | SDC |
| 65 | 4.11 | 4.084 | s | nucleoside derivatives, carbohydrates and amino acids | (1) | SDC |
| 66 | 4.084 | 4.077 | s | nucleoside derivatives, carbohydrates and amino acids | (1) | SDC |
| 67 | 4.077 | 4.067 | s | nucleoside derivatives, carbohydrates and amino acids | (1) | SDC |
| 68 | 4.067 | 4.057 | s | nucleoside derivatives, carbohydrates and amino acids | (1) | SDC |
| 69 | 4.057 | 4.038 | s | nucleoside derivatives, carbohydrates and amino acids | (1) | SDC |
| 70 | 4.038 | 4.024 | s | nucleoside derivatives, carbohydrates and amino acids | (1) | SDC |

| | | | | | | |
|---|---|---|---|---|---|---|
| 71 | 4.024 | 4.006 | s | nucleoside derivatives, carbohydrates and amino acids | (1) | SDC |
| 72 | 4.006 | 3.991 | s | nucleoside derivatives, carbohydrates and amino acids | (1) | SDC |
| 73 | 3.981 | 3.955 | t | carbohydrates and amino acids | (1) | SDC |
| 74 | 3.955 | 3.939 | t | carbohydrates and amino acids | (1) | SDC |
| 75 | 3.932 | 3.912 | t | carbohydrates and amino acids | (1) | SDC |
| 76 | 3.912 | 3.893 | t | carbohydrates and amino acids | (1) | SDC |
| 77 | 3.893 | 3.867 | t | carbohydrates and amino acids | (1) | SDC |
| 78 | 3.867 | 3.84 | t | carbohydrates and amino acids | (1) | SDC |
| 79 | 3.84 | 3.827 | t | carbohydrates and amino acids | (1) | SDC |
| 80 | 3.827 | 3.81 | t | carbohydrates and amino acids | (1) | SDC |
| 81 | 3.81 | 3.785 | t | carbohydrates and amino acids | (1) | SDC |
| 82 | 3.785 | 3.755 | t | carbohydrates and amino acids | (1) | SDC |
| 83 | 3.753 | 3.738 | t | carbohydrates and amino acids | (1) | SDC |
| 84 | 3.729 | 3.721 | t | carbohydrates and amino acids | (1) | SDC |
| 85 | 3.721 | 3.706 | t | carbohydrates and amino acids | (1) | SDC |
| 86 | 3.706 | 3.688 | t | carbohydrates and amino acids | (1) | SDC |
| 87 | 3.688 | 3.672 | t | carbohydrates and amino acids | (1) | SDC |
| 88 | 3.672 | 3.658 | t | carbohydrates and amino acids | (1) | SDC |
| 89 | 3.658 | 3.635 | t | carbohydrates and amino acids | (1) | SDC |
| 90 | 3.635 | 3.619 | t | carbohydrates and amino acids | (1) | SDC |
| 91 | 3.59 | 3.582 | u | carbohydrates | (1) | CAH |
| 92 | 3.582 | 3.573 | u | carbohydrates | (1) | CAH |
| 93 | 3.573 | 3.562 | u | carbohydrates | (1) | CAH |
| 94 | 3.562 | 3.553 | u | carbohydrates | (1) | CAH |
| 95 | 3.553 | 3.539 | u | carbohydrates | (1) | CAH |
| 96 | 3.476 | 3.451 | u | carbohydrates | (1) | CAH |
| 97 | 3.333 | 3.323 | u | carbohydrates | (1) | CAH |
| 98 | 3.323 | 3.312 | u | carbohydrates | (1) | CAH |
| 99 | 3.309 | 3.299 | u | carbohydrates | (1) | CAH |
| 100 | 3.269 | 3.26 | u | carbohydrates | (1) | CAH |
| 101 | 3.26 | 3.255 | u | carbohydrates | (1) | CAH |
| 102 | 3.255 | 3.209 | u | carbohydrates | (1) | CAH |
| 103 | 3.209 | 3.195 | | | | |
| 104 | 3.195 | 3.137 | | | | |
| 105 | 3.137 | 3.091 | | | | |
| 106 | 3.091 | 3.033 | v | ornithine | (1) TOCSY | OM |
| 107 | 3.033 | 2.92 | w | main putrescine, minor cadaverine and ornithine | (1) TOCSY | OM |
| 108 | 2.761 | 2.756 | | | | |
| 109 | 2.694 | 2.686 | | | | |
| 110 | 2.686 | 2.672 | | | | |
| 111 | 2.672 | 2.658 | | | | |
| 112 | 2.658 | 2.646 | | | | |
| 113 | 2.63 | 2.617 | x | succinate | (1) No correlation on TOCSY | OA |
| 114 | 2.563 | 2.555 | | | | |

| | | | | | | |
|---|---|---|---|---|---|---|
| 115 | 2.555 | 2.543 | | | | |
| 116 | 2.521 | 2.257 | | | | |
| 117 | 2.412 | 2.402 | | | | |
| 118 | 2.402 | 2.388 | | | | |
| 119 | 2.373 | 2.362 | | | | |
| 120 | 2.215 | 2.19 | y | oxaloacetate | (1) No correlation on TOCSY | OA |
| 121 | 2.19 | 2.163 | | | | |
| 122 | 2.083 | 2.076 | | | | |
| 123 | 2.076 | 2.058 | | | | |
| 124 | 2.058 | 2.043 | z | ornithine [f] | (1) TOCSY | OM |
| 125 | 2.043 | 2.03 | aa | Mainly acetate [e], also ornithine | Fan, BBA 1986 | OA |
| 126 | 2.03 | 2.012 | z | ornithine [f] | (1) TOCSY | OM |
| 127 | 2.012 | 1.994 | z | ornithine [f] | (1) TOCSY | OM |
| 128 | 1.994 | 1.978 | | | | |
| 129 | 1.978 | 1.973 | | | | |
| 130 | 1.973 | 1.963 | | | | |
| 131 | 1.963 | 1.95 | | | | |
| 132 | 1.95 | 1.944 | | | | |
| 133 | 1.944 | 1.931 | | | | |
| 134 | 1.931 | 1.92 | | | | |
| 135 | 1.92 | 1.91 | | | | |
| 136 | 1.91 | 1.899 | | | | |
| 137 | 1.865 | 1.85 | | | | |
| 138 | 1.85 | 1.824 | | | | |
| 139 | 1.752 | 1.742 | | | | |
| 140 | 1.742 | 1.728 | | | | |
| 141 | 1.728 | 1.717 | | | | |
| 142 | 1.717 | 1.676 | ab | Mainly putrescine, also ornithine | (1) TOCSY | OM |
| 143 | 1.676 | 1.661 | ac | arginine, lysine and cadaverine | (1) TOCSY | SDC |
| 144 | 1.661 | 1.648 | ac | arginine, lysine and cadaverine | (1) TOCSY | SDC |
| 145 | 1.648 | 1.633 | ac | arginine, lysine and cadaverine | (1) TOCSY | SDC |
| 146 | 1.633 | 1.617 | ac | arginine, lysine and cadaverine | (1) TOCSY | SDC |
| 147 | 1.55 | 1.538 | | | | |
| 148 | 1.538 | 1.514 | | | | |
| 149 | 1.514 | 1.499 | | | | |
| 150 | 1.499 | 1.477 | | | | |
| 151 | 1.425 | 1.41 | ad | *sharp signals*: arginine, lysine and cadaverine | (1) TOCSY | SDC [a] |
| 152 | 1.41 | 1.397 | ad | *sharp signals*: arginine, lysine and cadaverine | (1) TOCSY | SDC [a] |
| 153 | 1.4 | 1.3 | ae | *Broad* signals: lactate [g] | (1) TOCSY | OA |
| 154 | 1.397 | 1.382 | af | *sharp signals*: arginine, lysine and cadaverine | (1) TOCSY | SDC [a] |

| | | | | | | |
|---|---|---|---|---|---|---|
| 155 | 1.382 | 1.369 | af | *sharp signals*: arginine, lysine and cadaverine | (1) TOCSY | SDC [a] |
| 156 | 1.369 | 1.356 | af | *sharp signals*: arginine, lysine and cadaverine | (1) TOCSY | SDC [a] |
| 157 | 1.234 | 1.227 | | | | |
| 158 | 1.227 | 1.219 | | | | |
| 159 | 1.219 | 1.208 | | | | |
| 160 | 1.201 | 1.186 | | | | |
| 161 | 1.186 | 1.173 | | | | |
| 162 | 1.173 | 1.149 | | | | |
| 163 | 1.149 | 1.137 | | | | |
| 164 | 1.137 | 1.129 | | | | |
| 165 | 1.129 | 1.123 | | | | |
| 166 | 1.03 | 1.019 | ag | valine | (1) TOCSY | AA |
| 167 | 1.019 | 1.008 | ag | valine | (1) TOCSY | AA |
| 168 | 1.008 | 0.995 | ag | valine | (1) TOCSY | AA |
| 169 | 0.995 | 0.984 | | | | |
| 170 | 0.984 | 0.973 | | | | |
| 171 | 0.96 | 0.72 | | | | |
| 172 | 0.942 | 0.845 | ah | *broad signal*: fatty acids [g] | (1) TOCSY | OM |
| 173 | 0.845 | 0.717 | ah | *broad signal*: fatty acids [g] | (1) TOCSY | OM |
| 174 | 0.936 | 0.925 | ai | leucine | (1) TOCSY [h] | AA |
| 175 | 0.925 | 0.912 | ai | leucine | (1) TOCSY [h] | AA |
| 176 | 0.912 | 0.897 | ai | leucine | (1) TOCSY [h] | AA |
| 177 | 0.837 | 0.823 | | | | |
| 178 | 0.823 | 0.81 | | | | |
| 179 | 0.119 | 0.113 | | | | |
| 180 | 0.109 | 0.093 | | | | |

For each area: number, maximum and minimum chemical shift, in ppm, letter for the assignment on figure 2, assignment, assignment method, association of metabolite to their class. a: the area integrate the sharp signals. b: correlations visible on the TOCSY spectrum for other protons of the molecule in crowded regions, whose weak intensity is similar to that observed for down-field shifted diagonal signal. c: intense correlations visible on the TOCSY spectrum for other protons of the molecule. d: a triplet with J=52.3 Hz, due to coupling with $^{14}N$. A triplet upfield shifted by 0.02 ppm, ca. 35% of the intensity of the other triplet, assigned to $NH_3D^+$ due to the 10% $D_2O$ added to the sample. e: tentatively assigned on the basis of reference (2), and the absence of cross-peak wit this intense peak. f: a broad signal is also assigned to fatty acids. g: the broad signal dominates the area. h: assignment based on TOCSY cross-peaks. Other signals are weak compared with other at the same chemical shift.

Classes: NNA: nucleosides, nucleotides and analogues; AA: amino acids; CAH: carbohydrates; OA: organic acids; OM: other metabolites; SDC: superimposition of different classes.

(1) Fan TW-M, Lane AN. Structure-based profiling of metabolites and isotopomers by NMR. 2008, Prog Nucl Magn Reson Spectrosc; 52:69–117.

(2) Fan TW-M, Higashi RM, Lane AN, Jardetzky O. Combined use of 1H-NMR and GC-MS for metabolite monitoring and in vivo 1H-NMR assignments. 2008, Structure-based profiling of metabolites and isotopomers by NMR. Biochim Biophys Acta; 882:154-67.
